# Supplementary material for: The polymorphism of Hydra microsatellite sequences provides strain-specific signatures
Source: PLoS One. 2020 Sep 28;15(9):e0230547. doi: 10.1371/journal.pone.0230547 (PMC7521734; doi:10.1371/journal.pone.0230547)
Supplement: S1 Table — (DOCX) [file pone.0230547.s001.docx]

### S1 Table. Accession numbers of the *16S* ribosomal RNA and *Cytochrome C Oxydase I (COI)* sequences

*H. vulgaris-Pallas* strains were collected in Asia and Europe; *H. vulgaris-NA* strains were collected in North-America (*H. carnea, H. littoralis*) or derived from them as *AEP1* and *AEP2*. Sequences MN9886xy and MT0242xy are accessible at the URL: www.ncbi.nlm.nih.gov/genbank/.

| Gene name | Species | Strain | Accession |
| --- | --- | --- | --- |
| ***16S*** | *H. vulgaris-Pallas* | *Basel1* | MN988641 |
|  |  | *Basel2* | MN988642 |
|  |  | *Hm-105* | MN988634 |
|  |  | *reg-16* | MN988633 |
|  | *H. vulgaris-NA* | *AEP1* | MN988635 |
|  |  | *AEP2* | MN988636 |
|  | *H. oligactis* | *Ho_CR* | MN988639 |
|  |  | *Ho_CS* | MN988640 |
|  | *H. viridissima* | *Nicolet-Geneva* | MN988638 |
| ***COI*** | *H. vulgaris-Pallas* | *Basel1* | MT024257 |
|  |  | *Basel2* | MT024258 |
|  |  | *Hm-105* | MT024251 |
|  |  | *reg-16* | MT024252 |
|  | *H. vulgaris-NA* | *AEP1* | MT024253 |
|  |  | *AEP2* | MT024254 |
|  | *H. oligactis* | *Ho_CR* | MT024255 |
|  |  | *Ho_CS* | MT024256 |
|  | *H. viridissima* | *Nicolet-Geneva* | MT024260 |
