# Supplementary material for: The polymorphism of Hydra microsatellite sequences provides strain-specific signatures
Source: PLoS One. 2020 Sep 28;15(9):e0230547. doi: 10.1371/journal.pone.0230547 (PMC7521734; doi:10.1371/journal.pone.0230547)
Supplement: S2 Table — (DOCX) [file pone.0230547.s002.docx]

### S2 Table. Accession numbers of the *ms-c25145, ms-AIP, ms-DMTF1* microsatellite sequences.

The *Hydra 2.0* genomic sequences (34), the Juliano Trinity and Juliano aepLRv2 transcriptomic sequences (38) are available at the [NHGRI *Hydra* web portal](https://research.nhgri.nih.gov/hydra/databases.shtml), the *AEP ecto-GFP* (40) and *H. oligactis* sequences (32)(41) at [HydrATLAS](https://hydratlas.unige.ch/), the *HAEP* (43) and *Hm-105* (37) transcriptomic sequences at [Compagen](http://www.compagen.org/); the *Hv-Basel1* reference transcriptome is available at [Uniprot](https://www.uniprot.org/uniprot/?query=hydra+vulgaris&sort=score) (35,36). “AEP” indicates that the strain was not characterized. Sequences MT024xyz from this study are available at: [www.ncbi.nlm.nih.gov/genbank/](http://www.ncbi.nlm.nih.gov/genbank/).

|  | Species | Strain | Databases | Accession number | Nb of polyps sequenced |
| --- | --- | --- | --- | --- | --- |
| ***ms-c25145*** | ***H. vulgaris-Pallas*** | *Basel1* | This study (genomic) | MT024275 | 3 polyps (8 colonies) |
|  |  |  |  | MT024277 | 3 polyps (5 colonies) |
|  |  |  |  | MT024278 | 1 polyp |
|  |  | *Basel2* | This study (genomic) | MT024276 | 2 polyps (2 colonies) |
|  |  | *Hm-105* | *Hydra* 2.0 genome | Sc4wPfr_396.1 | - |
|  |  |  |  | Sc4wPfr_1246 | - |
|  |  |  | Juliano Trinity | not detected | |
|  |  |  | Compagen | not detected | |
|  |  |  | This study (genomic) | MT024273 | 5 polyps (24 colonies) |
|  |  |  |  | MT024274, MT024279 | 2 distinct polyps |
|  |  |  |  | MT024280 | 5 polyps (10 colonies) |
|  | ***H. vulgaris-NA*** | *AEP ecto-GFP* | HydrATLAS | c25145_g1_i09 | Stem cell RNA-seq |
|  |  | *AEP* | Juliano aepLRv2 | not detected | |
|  |  |  | Compagen | HAEP_T-CDS_v02_11692 | - |
|  |  | *AEP1* | This study (genomic) | MT024265, MT024266,  MT024269, MT024271 | 4 distinct polyps |
|  |  |  |  | MT024270 | 1 polyp (2 colonies) |
|  |  | *AEP2* | This study (genomic) | MT024267, MT024268 | 2 distinct polyps |
|  |  |  |  | MT024272 | 4 polyps (6 colonies) |
|  | ***H. oligactis*** | *Ho_CR* | *Ho* genome draft | CR_jcf7180012595266 | - |
|  |  |  | HydrATLAS | R039447c0g1_i05 (MT024261) | *Ho_CR* RNA-seq |
|  |  |  | This study (genomic) | MT024262 | 2 polyps (2 colonies) |
|  |  |  |  | MT024264 | 1 polyp |
|  |  | *Ho_CS* | HydrATLAS | not detected | |
|  |  |  | This study (genomic) | MT024263 | 3 polyps (4 colonies) |
|  |  |  |  |  |  |
| ***ms-AIP*** | ***H. vulgaris-Pallas*** | *Basel1* | This study (genomic) | MT024287 | 1 polyp |
|  |  | *Basel2* | This study (genomic) | MT024292 | 1 polyp |
|  |  | *Hm-105* | *Hydra* 2.0 genome | lcl\|Sc4wPfr_417.3 (MT024295) | - |
|  |  |  | Juliano Trinity | JT_TRINITY_DN8247_c0_g1_i1 (MT024291) | - |
|  |  |  | Compagen | not detected | |
|  |  |  | This study (genomic) | MT024289, MT024290,  MT024293, MT024294 | 4 distinct polyps |
|  |  | *reg-16* | This study (genomic) | MT024288 | 1 polyp |
|  | ***H. vulgaris-NA*** | *AEP ecto-GFP* | HydrATLAS | c8134_g1_i01 | Stem cell RNA-seq |
|  |  | *AEP* | Juliano aepLRv2 | lcl\|t9630aep | - |
|  |  |  | Compagen | HAEP_T-CDS_v02_12771 (MT024285) | - |
|  |  | *AEP1* | This study (genomic) | MT024281, MT024283 | 2 distinct polyps |
|  |  | *AEP2* | This study (genomic) | MT024282, MT024284, MT024286 | 3 distinct polyps |
|  |  |  |  |  |  |
| ***ms-DMTF1*** | ***H. vulgaris-Pallas*** | *Hm-105* | *Hydra* 2.0 genome | lcl\|Sc4wPfr_417.3 |  |
|  | ***H. vulgaris-NA*** | *AEP ecto-GFP* | HydrATLAS | c21737_g1_i04 | Stem cell RNA-seq |
|  |  | *AEP ecto-GFP* | This study (genomic) | MT024299 | 1 polyp |
|  |  | *AEP endo-GFP* | This study (genomic) | MT024296 | 1 polyp |
|  |  | *AEP* | Juliano aepLRv2 | lcl\|t12151aep | - |
|  |  |  | Compagen | HAEP_T-CDS_v02_6413 | - |
|  |  | *AEP2* | This study (genomic) | MT024300 | 1 polyp |
|  |  | *AEP2 Q82-*203 | This study (genomic) | MT024297 | 1 polyp (2 colonies) |
|  |  | *AEP2 Q82*-293 | This study (genomic) | MT024298 | 1 polyp (2 colonies) |
