# Supplementary material for: The polymorphism of Hydra microsatellite sequences provides strain-specific signatures
Source: PLoS One. 2020 Sep 28;15(9):e0230547. doi: 10.1371/journal.pone.0230547 (PMC7521734; doi:10.1371/journal.pone.0230547)
Supplement: S1 Fig — (DOCX) [file pone.0230547.s003.docx]

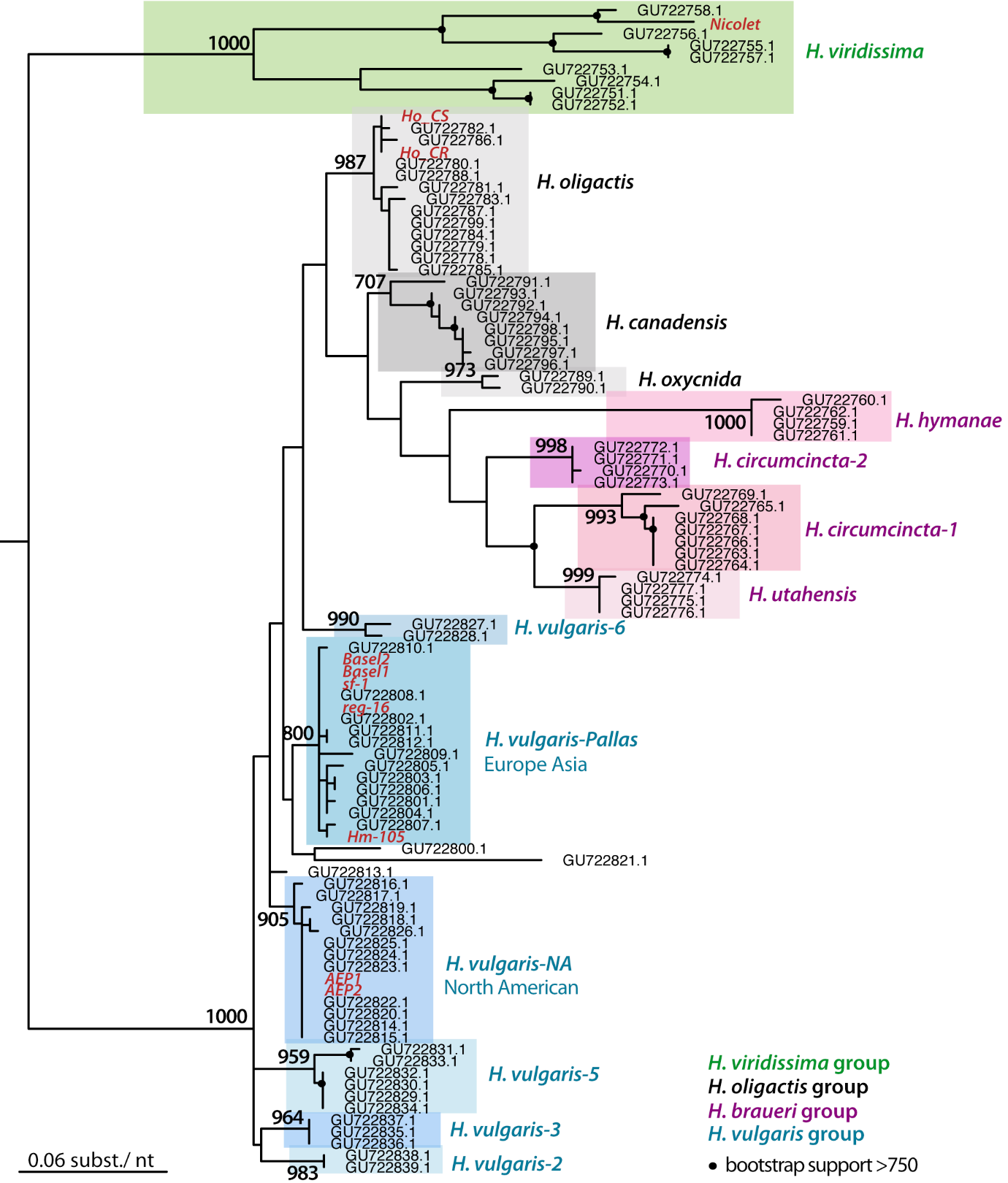


### S1 Fig. Phylogenetic reconstruction of the *Hydra* genus based on the analysis of the 16S ribosomal RNA sequences.

The maximum likelihood (ML) tree of the *16S* ribosomal RNA sequences was built by adding to the 89 sequences dataset available on Genbank (Martinez et al., 2010) nine sequences obtained in the present study (written in red, see **S2 Table** for accession numbers). The sequences were aligned with ClustalW (BioEdit) and the tree was built after performing a ML 3.0 analysis using the GTR substitution model. The robustness of the nodes was deduced from 1’000 bootstraps. This tree confirms the presence of six sub-group/species in the *H. vulgaris* group. Note the position of the *Basel, reg-16, sf-1* and *Hm-105* sequences in the *H. vulgaris-Pallas* sub-group, and the *AEP1* and *AEP2* sequences within the *H. vulgaris-NA* sub-group.
