# Supplementary material for: The polymorphism of Hydra microsatellite sequences provides strain-specific signatures
Source: PLoS One. 2020 Sep 28;15(9):e0230547. doi: 10.1371/journal.pone.0230547 (PMC7521734; doi:10.1371/journal.pone.0230547)
Supplement: S2 Fig — (DOCX) [file pone.0230547.s004.docx]

#### Alignment of the Hydra genomic and transcriptomic ms_c25145 sequences

#### >c25145_Hm105, 274 AAs long, Hm-105_lcl|Sc4wPfr_396.1, Hydra C25145_g1_i04

MFYIVHFLNDNTVEYVPKEWLNGNSEC**M**WPKCSMTSLKGMRRKRQIPNKDWERYKIRILSTADCEERALEKLKISEETSDLVSEYEGNSCHRKTTSKRLSPSLFASQIHGNMSSEDDSDFNMPTSLQQAVISTSTPGSQPLLPIPHHANVFREMQHQGKSSQSFVSLLNDPDEINLPSLYQEFLPIPYTKNSTNTLTPITQSIEYRLSSIENLLKDLVKSVTSATKEIKHLIERMPIGHTEESLFTKSSSIEELDAVLLQCQDEEMASLFASFT

10 20 30 40 50 60 70 80 90 100

....|....|....|....|....|....|....|....|....|....|....|....|....|....|....|....|....|....|....|....|

**C25145_g1_i04 M F Y I V H F L N D N T V E**

Hm-105_lcl|Sc4wPfr_396.1 ----------------------------------------------------------------------------------------------------

Hm-105_lcl|Sc4wPfr_1246 TTTTTTATAATCTGCACAAATAGTTTACATTATTTATATTTTTTACAGTGTTAAGATATGTTTTATATTGTTCATTTCCTTGACGACAGTACTGTGGAGT

AEP_c25145_g1_i09 ----------------------------------------------AGTGTAAAGATATGTTTTATATTGTTCATTTCCTTAACGACAATACTGTGGAGT

AEP_c25145_g1_i04 ----------------------------------------------AGTGTAAAGATATGTTTTATATTGTTCATTTCCTTAACGACAATACTGTGGAGT

AEP_HAEP_T-CDS_v02_11692 ------------------------------------------------TGTAAAGATATGTTTTATATTGTTCATTTCCTTAACGACAATACTGTGGAGT

AEP_c25145_g1_i07 ----------------------------------------------------------------------------------------------------

AEP_HAEP_T-CDS_v02_7716 -GACGGAAAAAAACACATACTGAAACTTTTTAGTCCCTGTGTAATAAGTGTAAAGATATGTTTTATATTGTTCATTTCCTTAACGACAATACTGTGGAGT

Ho_CS_S040478c1g2_i08 -------------------------GCTGTTCTTTTTGAACTCTTCTGTGTAAAGATATGTTTTATATTGTTCATTTCCTTAACGACAATACTGTGGAGT

Ho_CS_S040478c1g2_i03 -------------------------GCTGTTCTTTTTGAACTCTTCTGTGTAAAGATATGTTTTATATTGTTCATTTCCTTAACGACAATACTGTGGAGT

Ho_CR_R039447c0g1_i0 -----------------------------------------TTTACAGTGTAAAGATATGTTTTATATTGATCATTTCCTTAACGACAATACTGTGGAGT

Ho_CR_R039447c0g1_i2 -------------------TCCATATCCTTTTTCATTTATGCATATAGTGTAAAGATATGTTTTATATTGTTCATTTCCTTAACGACAATACTGTGGAGT

110 120 130 140 150 160 170 180 190 200

....|....|....|....|....|....|....|....|....|....|....|....|....|....|....|....|....|....|....|....|

**C25145_g1_i04 Y V P K E W L N G N S E C M W P K C S M T S L K G M R R K R Q I P N**

Hm-105_lcl|Sc4wPfr_396.1 --------------------AATGGAAACAGCGAGTGTATTTGACCAAAATGTAGCATCACTTCATTAAAAGGAATGCGCTGGAAGAGACAGATTCCCAA

Hm-105_lcl|Sc4wPfr_1246 ATGTTCCAAAGAAAGGGTTGAATAGAAACAGCGAGTGTATGTGGGCAAAATGTAGCATCACTTTATTAAAAGGAATGCACCGGAAGAGACAGATTCCCAA

AEP_c25145_g1_i09 ATGTTCCAAAGGAATGGCTTAATGGAAACAGCGAGTGTATGTGGCCAAAATGTAGCATGACTTCATTAAAAGGAATGCGCCGGAAGAGACAGATTCCCAA

AEP_c25145_g1_i04 ATGTTCCAAAGGAATGGCTTAATGGAAACAGCGAGTGTATGTGGCCAAAATGTAGCATGACTTCATTAAAAGGAATGCGCCGGAAGAGACAGATTCCCAA

AEP_HAEP_T-CDS_v02_11692 ATGTTCCAAAGGAATGGCCTAATGGAAACAGCGAGTGTATGTGGCCAAAATGTAGCATGACTTCATTAAAAGGAATGCGCCGGAAGAGACAGATTCCCAA

AEP_c25145_g1_i07 ------------------------------------------------AATGTAGCATGACTTCATTAAAAGGAATGCGCCGGAAGAGACAGATTCCCAA

AEP_HAEP_T-CDS_v02_7716 ATGTTCCAAAGGAATGGCTTAATGGAAACAGCGAGTGTATGTGGCCAAAATGTAGCATGACTTCATTAAAAGGAATGCGCCGGAAGAGACAGATTCCCAA

Ho_CS_S040478c1g2_i08 ATGTTCCAAAGGAATGGCTTAATGGAAACAGTGAGTGTATGTGGCCAAATTGTAGCATGACTTCATTAAAAGGAATGCGCCGGAAGAGACAGATTCCCAA

Ho_CS_S040478c1g2_i03 ATGTTCCAAAGGAATGGCTTAATGGAAACAGTGAGTGTATGTGGCCAAATTGTAGCATGACTTCATTAAAAGGAATGCGCCGGAAGAGACAGATTCCCAA

Ho_CR_R039447c0g1_i05 ATGTTCCAAAGGAATGGCTTAATG----TAGCGAGTGTATGTGGCCAAAATGTAGCATGACTTCATTAAAAGGAATGCGCCGGAAGAGACAGATTCCCAA

Ho_CR_R039447c0g1_i02 ATGTTCCAAAGGAATGGCTTAATGGAAACAGTGAGTGTATGTGGCCAAATTGTAGCATGACTTCATTAAAAGGAATGCGCCGGAAGAGACAGATTCCCAA

210 220 230 240 250 260 270 280 290 300

....|....|....|....|....|....|....|....|....|....|....|....|....|....|....|....|....|....|....|....|

**C25145_g1_i04 K D W E R Y K I R I L S T A**

Hm-105_lcl|Sc4wPfr_396.1 TAAAGATTAGGAAAGATAAAAAAATACGAATTTTATCTAATGCAGataaacacacatacatatatatgtatgtgtgtgtgtgtgtgtgtgtgtgtgtgtg

Hm-105_lcl|Sc4wPfr_1246 TAAAGATTGGGAAAGATATAAA-ATTAGTA--------------Gataaacacacacacatatat----------gtgtgtctgtgcgtgtgtgcgtgtg

AEP_c25145_g1_i09 TAAAGATTGGGAAAGATACAAA-ATACGAATTTTATCTACTGCAGgtaaacacacacatatg--------------tgtgtgtgtttgtgtgtgtgtttg

AEP_c25145_g1_i04 TAAAGATTGGGAAAGATACAAA-ATACGAATTTTATCTACTGCAGgtaaacacacacatatg--------------tgtgtgtgtttgtgtgtgtgtttg

AEP_HAEP_T-CDS_v02_11692 TAAAGATTGGGAAAGATACAAA-ATACGAATTTTATCTACTGCAGgtaaacacacacatatg--------------tgtgtgtgtttgtgtgtgtgtgtg

AEP_c25145_g1_i07 TAAAGATTGGGAAAGATACAAA-ATACGAATTTTATCTACTGCA--------------------------------------------------------

AEP_HAEP_T-CDS_v02_7716 TAAAGATTGGGAAAGATACAAA-ATACGAATTTTATCTACTGCA--------------------------------------------------------

Ho_CS_S040478c1g2_i08 TAAAGATTGG-AAAGATACAAA-ATACGAATTTTATCTACTGCA--------------------------------------------------------

Ho_CS_S040478c1g2_i03 TAAAGATTGG-AAAGATACAAA-ATACGAATTTTATCTACTGCA--------------------------------------------------------

Ho_CR_R039447c0g1_i05 TAAAGATTGGGAAAGATACAAA-ATACGAATTTTATCTACTGCAGgtaaacacacacatatg--------------tgtgtgtgtttgtgtatgtgtgtg

Ho_CR_R039447c0g1_i02 TAAAGATTGG-AAAGATACAAA-ATACGAATTTTATCTACTGCA--------------------------------------------------------

310 320 330 340 350 360 370 380 390 400

....|....|....|....|....|....|....|....|....|....|....|....|....|....|....|....|....|....|....|....|

Hm-105_lcl|Sc4wPfr_396.1 cacgcgcacgcatttaaattatacctggtttattaatattactttaatgcacaatcaaatcaagtttttgttacttctttttg----------------a

Hm-105_lcl|Sc4wPfr_1246 cgca--------tttaaattatgcctggtttattaatattactttaatgcataatcaaaccaaatttttgttacttctctttg----------------a

AEP_c25145_g1_i09 cg----------tttaagttatacctggtttattaatattactttaatgcacaatcaactcacatttttgttactttgtactactttgactactttgtta

AEP_c25145_g1_i04 cg----------tttaagttatacctggtttattaatattactttaatgcacaatcaactcacatttttgttactttgtactactttgactactttgtta

AEP_HAEP_T-CDS_v02_11692 cg----------tttaagttatacctggtttattaatattactttaatgcacaatcaactcacatttttgttactttgtactactttgactactttgtta

AEP_c25145_g1_i07 ----------------------------------------------------------------------------------------------------

AEP_HAEP_T-CDS_v02_7716 ----------------------------------------------------------------------------------------------------

Ho_CS_S040478c1g2_i08 ----------------------------------------------------------------------------------------------------

Ho_CS_S040478c1g2_i03 ----------------------------------------------------------------------------------------------------

Ho_CR_R039447c0g1_i05 cg----------tttaagttatacctggtttattaatattactttaatgcacaatcaactcaaatttttgttactttgtactactttgactactttgtta

Ho_CR_R039447c0g1_i02 ----------------------------------------------------------------------------------------------------

410 420 430 440 450 460 470 480 490 500

....|....|....|....|....|....|....|....|....|....|....|....|....|....|....|....|....|....|....|....|

**C25145_g1_i04 D C E E R A L E K L K I S E E T S D L V S E Y E G N S C H R K T T**

Hm-105_lcl|Sc4wPfr_396.1 cagACTGCGAGGAAAGAGCATTAGAAAAGTTAAAAATATCTGAAGAAACAAATGATCTTCCTTCAGTATATGAAGGTGATTCATGCCGTAGGAAAACAAC

Hm-105_lcl|Sc4wPfr_1246 cagACTGTGAGGAAAGAGCATTAGAAAAGTTAAAAACATCTGATGAAACAAGTGATCTTGCTTCAGCAAATGAAAGTGATTCATGTCGTAGAAAAACAAC

AEP_c25145_g1_i09 cagACTGTGAGGAAAGAGCATTAGAAAAGTTAAAAATATCTGAAGAAACAAGTGATCTTGCTTCAGAATATGAAGGTAATTCATGTCATAGGAAAACAAC

AEP_c25145_g1_i04 cagACTGTGAGGAAAGAGCATTAGAAAAGTTAAAAATATCTGAAGAAACAAGTGATCTTGCTTCAGAATATGAAGGTAATTCATGTCATAGGAAAACAAC

AEP_HAEP_T-CDS_v02_11692 cagACTGTGAGGAAAGAGCATTAGAAAAGTTAAAAATATCTGAAGAAACAAGTGATCTTGCTTCAGAATATGAAGGTAATTCATGTCATAGGAAACAA--

AEP_c25145_g1_i07 --gACTGTGAGGAAAGAGCATTAGAAAAGTTAAAAATATCTGAAGAAACAAGTGATCTTGCTTCAGAATATGAAGGTAATTCATGTCATAGGAAAACAAC

AEP_HAEP_T-CDS_v02_7716 --gACTGTGAGGAAAGAGCATTAGAAAAGTTAAAAATATCTGAAGAAACAAGTGATCTTGTTTCAGAATATGAAGGTAATTCATGTCATAGGAAAACAAC

Ho_CS_S040478c1g2_i08 --gACTGTGAGGAAAGAGCATTAGAAAAGTTAAAAATATCTGAAGAAACAAGTGATCTTGCTTCAGAATATGAAGGTAATTCATGTCGTAGGAAAACAAC

Ho_CS_S040478c1g2_i03 --gACTGTGAGGAAAGAGCATTAGAAAAGTTAAAAATATCTGAAGAAACAAGTGATCTTGCTTCAGAATATGAAGGTAATTCATGTCGTAGGAAAACAAC

Ho_CR_R039447c0g1_i05 cagACTGTGAGGAAAGAGCATTAGAAAAGTTAAAAATATCTGAAGAAACAAGTGATCTTGCTTCAGAATATGAAGGTAATTCATGCCGTATGAAATGAAC

Ho_CR_R039447c0g1_i02 --gACTGTGAGGAAAGAGCATTAGAAAAGTTAAAAATATCTGAAGAAACAAGTGATCTTGCTTCAGAATATGAAGGTAATTCATGTCGTAGGAAAACAAC

510 520 530 540 550 560 570 580 590 600

....|....|....|....|....|....|....|....|....|....|....|....|....|....|....|....|....|....|....|....|

**C25145_g1_i04 S K R L S P S L F A S Q I H G N M S S E D D S D F N M P T S L Q Q**

Hm-105_lcl|Sc4wPfr_396.1 GTATAAACGATTATCCGTATTCATTATTTGTTTCTGAAACTCATGGCAATATGTATAGTGAAGATGATTTTGATTTCAACATGCCTTCTTCTCTTCAGCC

Hm-105_lcl|Sc4wPfr_1246 GTCTAAACGATTATCC-CCTTCATTATTTGCTTCTTAAATTCATAGCAATATGTATAGTGAAGATGGTTCTGATTTCAACATGCCTTCATCTCTTCAGCC

AEP_c25145_g1_i09 ATCAAAACGATTATCC-CCTTCATTATTTGCTTCTCAAATTCATGGCAATATGTCTAGTGAAGATGATTCTGATTTCAACATGCCTACATCTCTTCAGCA

AEP_c25145_g1_i04 ATCAAAACGATTATCC-CCTTCATTATTTGCTTCTCAAATTCATGGCAATATGTCTAGTGAAGATGATTCTGATTTCAACATGCCTACATCTCTTCAGCA

AEP_HAEP_T-CDS_v02_11692 ----------------------------------------------------------------------------------------------------

AEP_c25145_g1_i07 ATCAAAACGATTATCC-CCTTCATTATTTGCTTCTCAAATTCATGGCAATATGTCTAGTGAAGATGATTCTGATTTCAACATGCCTACATCTCTTCAGCA

AEP_HAEP_T-CDS_v02_7716 ATCAAAACGATTATCC-CCTTCATTATTTGCTTCTCAAATTCATGGCAATATGTCTAGTGAAGATGATTCTGATTTCAACATGCCTACATCTCTTCAGCA

Ho_CS_S040478c1g2_i08 ATCAAAACGATTATCC-CCTTCATTATTTGCTTCTCAAATTCATGGCAATATGTCTAGTGAAGATGATTCTGATTTCAACATGCCTACATCTCTTCAGAC

Ho_CS_S040478c1g2_i03 ATCAAAACGATTATCC-CCTTCATTATTTGCTTCTCAAATTCATGGCAATATGTCTAGTGAAGATGATTCTGATTTCAACATGCCTACATCTCTTCAGAC

Ho_CR_R039447c0g1_i05 -------------------------------------AATTCATGGCA--ATGTCTAGTGAAGATGATTCTGATTTCAACATGCCTACTTCTCTTCAGCA

Ho_CR_R039447c0g1_i02 ATCAAAACGATTATCC-CCTTCATTATTTGCTTCTCAAATTCATGGCAATATGTCTAGTGAAGATGATTCTGATTTCAACATGCCTACATCTAATCAGCC

#### Alignment of the Hydra genomic and transcriptomic ms_c25145 sequences

610 620 630 640 650 660 670 680 690 700

....|....|....|....|....|....|....|....|....|....|....|....|....|....|....|....|....|....|....|....|

**C25145_g1_i04 A V I S**

Hm-105_lcl|Sc4wPfr_396.1 AGCTGTCATTTCTgtaagcttataaagtgacttcattaaaaacaagtgtaaacaagttaatgaaaaagtttttaaataattgttttaaatgtggaaatga

Hm-105_lcl|Sc4wPfr_1246 AGCTGTCATTTCTataagcttataaagtggctttattaaaaataagtgtaaaaaatgttggaaaagcaaacttccatttgcttttccaacatatcctgct

AEP_c25145_g1_i09 GGCTGTCATTTCT---------------------------------------------------------------------------------------

AEP_c25145_g1_i04 GGCTGTCATTTCT---------------------------------------------------------------------------------------

AEP_HAEP_T-CDS_v02_11692 ----------------------------------------------------------------------------------------------------

AEP_c25145_g1_i07 GGCTGTCATTTCT---------------------------------------------------------------------------------------

AEP_HAEP_T-CDS_v02_7716 GGCTGTCATTTCT---------------------------------------------------------------------------------------

Ho_CS_S040478c1g2_i08 AGCTGTCATTTCT---------------------------------------------------------------------------------------

Ho_CS_S040478c1g2_i03 AGCTGTCATTTCT---------------------------------------------------------------------------------------

Ho_CR_R039447c0g1_i05 GGCTGTCATTTCT---------------------------------------------------------------------------------------

Ho_CR_R039447c0g1_i02 GGCTGTCATTTCT---------------------------------------------------------------------------------------

Hm-105_lcl|Sc4wPfr_396.1 aaatatttatttaaacatggacttacacttattgattacaatcaaaaaatatatatttttttaaattagttaaaattactactaaaattactagatttta 800

Hm-105_lcl|Sc4wPfr_1246 agtgcaggatatgttggaaaagcaaatggaagaagaagtctttacttgttggggtaccctctccaaatattttctccaaaaaaacttccaaccggtgggg 800

810 820 830 840 850 860 870 880 890 900

....|....|....|....|....|....|....|....|....|....|....|....|....|....|....|....|....|....|....|....|

**C25145_g1_i04 T S T P G S Q P L L P I P H H A**

Hm-105_lcl|Sc4wPfr_396.1 ttaattaacataaaattattttagACATCAACACCTAGTAGCCAACCATTACTGCCAATACCACAACATGGTgtaagtattaaacatgatgcttgatagc

Hm-105_lcl|Sc4wPfr_1246 aagtttgtcgaagaatttactgga----------------------------------------------------------------------------

AEP_c25145_g1_i09 ------------------------ACATCAACACCAGGTAGCCAACCATTACTGCCAATACCACATCATGCT----------------------------

AEP_c25145_g1_i04 ------------------------ACATCAACACCAGGTAGCCAACCATTACTGCCAATACCACACCATGCT----------------------------

AEP_HAEP_T-CDS_v02_11692 ----------------------------------------------------------------------------------------------------

AEP_c25145_g1_i07 ------------------------ACATCAACACCAGGTAGCCAACCATTACTGCCAATACCACATCATGCT----------------------------

AEP_HAEP_T-CDS_v02_7716 ------------------------ACATCAACACCAGGTAGCCAACCATTACTGCCAATACCACATCATGCT----------------------------

Ho_CS_S040478c1g2_i08 ------------------------ACATCAACACCTAGTAGCCAACCATTACTGCCAATACCACATTATGCT----------------------------

Ho_CS_S040478c1g2_i03 ------------------------ACATCAACACCTAGTAGCCAACCATTACTGCCAATACCACATTATGCT----------------------------

Ho_CR_R039447c0g1_i05 ------------------------ACATCAACCCCTAGTAGCCAACCATTACTGCCAATACCACATCATGCT----------------------------

Ho_CR_R039447c0g1_i02 ------------------------ACATCAACACCTAGTAGCCAACCATTACTGCCAATACCACATCATGCT----------------------------

910 920 930 940 950 960 970 980 990 1000

....|....|....|....|....|....|....|....|....|....|....|....|....|....|....|....|....|....|....|....|

**C25145_g1_i04 N V F R E M Q H Q G K S S**

Hm-105_lcl|Sc4wPfr_396.1 aatactttaagttaaaatttgttaatgttactattaggtattaaaatttgataaatgattagAATGTTTTTCGAGAGATCCAACACCAAGACAAATCTTG

Hm-105_lcl|Sc4wPfr_1246 ----------------------------------------------------------------------------------------------------

AEP_c25145_g1_i09 --------------------------------------------------------------AATGTTTTTCGAGAGATGCAACACCAAGGAAAATCTAG

AEP_c25145_g1_i04 --------------------------------------------------------------ATT-----------------------------------

AEP_HAEP_T-CDS_v02_11692 ----------------------------------------------------------------------------------------------------

AEP_c25145_g1_i07 --------------------------------------------------------------AATGTTTTTCGAGAGATGCAACACCAAGGAAAATCTAG

AEP_HAEP_T-CDS_v02_7716 --------------------------------------------------------------AATGTTTTTCGAGAGATGCAACACCAAGGAAAATCTAG

Ho_CS_S040478c1g2_i08 --------------------------------------------------------------AATGTTTTTCGAGAGATGCAACACCAAGGAAAATCTAG

Ho_CS_S040478c1g2_i03 --------------------------------------------------------------AATGTTTTTCGAGAGATGCAACACCAAGGAAAATCTAG

Ho_CR_R039447c0g1_i05 --------------------------------------------------------------AATGTTTTTCGAGAGATGCAACACCAAGGAAAATCTAG

Ho_CR_R039447c0g1_i02 --------------------------------------------------------------AATGTTTTTCGAGAGATGCAACACCAAGGAAAATCTAG

1010 1020 1030 1040 1050 1060 1070 1080 1090 1100

....|....|....|....|....|....|....|....|....|....|....|....|....|....|....|....|....|....|....|....|

**C25145_g1_i04 Q S F V S L L N D P D E I N L P S**

Hm-105_lcl|Sc4wPfr_396.1 TCAAAGCTTCGTATCATTACCAAATGATCCTGAAGAAATAAATTTACCTTCAgtaagctttttgtaaattttattttagtaattatatgtttgctgttaa

Hm-105_lcl|Sc4wPfr_1246 ----------------------------------------------------------------------------------------------------

AEP_c25145_g1_i09 TCAAAGCTTTGTGTCATTACTAAATGATCCTGATGAAATAAATTTACCTTCA------------------------------------------------

AEP_c25145_g1_i04 ----------------------------------------------------------------------------------------------------

AEP_HAEP_T-CDS_v02_11692 ----------------------------------------------------------------------------------------------------

AEP_c25145_g1_i07 TCAAAGCTTTGTGTCATTACTAAATGATCCTGATGAAATAAATTTACCTTCA------------------------------------------------

AEP_HAEP_T-CDS_v02_7716 TCAAAGCTTTGTGTCATTACTAAATGATCCTGATGAAATAAATTTACCTTCA------------------------------------------------

Ho_CS_S040478c1g2_i08 TCAAAGCTTTGTGTCATTACTAAATGATCCTGATGAAATAAATTTACCTTCA------------------------------------------------

Ho_CS_S040478c1g2_i03 TCAAAGCTTTGTGTCATTACTAAATGATCCTGATGAAATAAATTTACCTTCA------------------------------------------------

Ho_CR_R039447c0g1_i05 TCAAAGCTTTGTGTCATTACTAAATGATCCTGATGAATCAAATTTACTTTCA------------------------------------------------

Ho_CR_R039447c0g1_i02 TCAAAGCTTTGTGTCATTACTAAATGATCCTGATGAATCAAATTTACTTTCA------------------------------------------------

Hm-105_lcl|Sc4wPfr_396.1 gtaaaataattataattaacgatcattaaattaaaatatgcatagatgcaaattaaactagaaaattatatcttttaaattgtgtatgttgatattttaa

Hm-105_lcl|Sc4wPfr_1246 ----------------------------------------------------------------------------------------------------

1210 1220 1230 1240 1250 1260 1270 1280 1290 1300

....|....|....|....|....|....|....|....|....|....|....|....|....|....|....|....|....|....|....|....|

**C25145_g1_i04 L Y Q E F L P I P Y T K N S T N T L T P I T Q S**

Hm-105_lcl|Sc4wPfr_396.1 taatataattatactatattttgttggagTTATCTCAGAAGTTCTTGCCAATCCCTCACACCGAAAACTCCACCTACACCCTTACACCAATCACACAGTC

Hm-105_lcl|Sc4wPfr_1246 ----------------------------------------------------------------------------------------------------

AEP_c25145_g1_i09 ----------------------------------------------------------------------------------------------------

AEP_c25145_g1_i04 ----------------------------------------------------------------------------------------------------

AEP_HAEP_T-CDS_v02_11692 ----------------------------------------------------------------------------------------------------

AEP_c25145_g1_i07 ----------------------------------------------------------------------------------------------------

AEP_HAEP_T-CDS_v02_7716 -----------------------------TTATATCAGGAGTTCTTGCCAATCCCATACACCAAAAATTCCACTAACACCCTTACACCAATCACACAGTC

Ho_CS_S040478c1g2_i08 --------------------------------------GAGTTCTTGCCAATCCCATACACCAAAAATTCCACTAACACCCTTCCACCAATCACACAGTC

Ho_CS_S040478c1g2_i03 --------------------------------------GAGTTCTTGCCAATCCCATACACCAAAAATTCCACTAACACCCTTCCACCAATCACACAGTC

Ho_CR_R039447c0g1_i05 -----------------------------TTATCTCAGGAGTTCTTGCCAATCCCATACACCAAAAATTCCACTAACACCCTTACACCAATCACACAGTC

Ho_CR_R039447c0g1_i02 --------------------------------------GAGTTCTTGCCAATCCCATACACCAAAAATTCCACTAACACCCTTACACCAATCACACAGTC

1310 1320 1330 1340 1350 1360 1370 1380 1390 1400

....|....|....|....|....|....|....|....|....|....|....|....|....|....|....|....|....|....|....|....|

**C25145_g1_i04 I E Y R L S S I E N L L K D**

Hm-105_lcl|Sc4wPfr_396.1 AATTGAATACCGATTTTCTTACATTGAAAACCTACTTAAAGATgaaaaatcttttaaagtacattaatttgtctttcttttttatacttgtaaaagttga

Hm-105_lcl|Sc4wPfr_1246 ----------------------------------------------------------------------------------------------------

AEP_c25145_g1_i09 ----------------------------------------------------------------------------------------------------

AEP_c25145_g1_i04 ----------------------------------------------------------------------------------------------------

AEP_HAEP_T-CDS_v02_11692 ----------------------------------------------------------------------------------------------------

AEP_c25145_g1_i07 ----------------------------------------------------------------------------------------------------

AEP_HAEP_T-CDS_v02_7716 AATTGAATACCGATTGTCCTCCATTGAAAATTTACTTAAAGAT---------------------------------------------------------

Ho_CS_S040478c1g2_i08 AATTGAATACCGATTGTCCTCCATTGAAAATTTACTTAAAGGTgaaaaatcttttaaagtacatatattaatttgtctatcttttaaatacttataacag

Ho_CS_S040478c1g2_i03 AATTGAATACCGATTGTCCTCCATTGAAAATTTACTTAAAGAT---------------------------------------------------------

Ho_CR_R039447c0g1_i05 AATTGAATACCGATTGTCCTCCATTGAAAATTTACTTAAAGAT---------------------------------------------------------

Ho_CR_R039447c0g1_i02 AATTGAATACCGATTGTCCTCCATTGAAAATTTACTTAAAGAT---------------------------------------------------------

#### Alignment of the Hydra genomic and transcriptomic ms_c25145 sequences

1410 1420 1430 1440 1450 1460 1470 1480 1490 1500

...|....|....|....|....|....|....|....|....|....|....|....|....|....|....|....|....|....|....|....|

**C25145_g1_i04 L V K S V T S A T K E I K H L I**

Hm-105_lcl|Sc4wPfr_396.1 tttgattaaat-----TTAGTAAAATCTGTAACTTCTGCGACAAAAGAGATTAAGAGCGTATGCCAATTGgtcatactgaagagacagagcgtatgccaa

Hm-105_lcl|Sc4wPfr_1246 ----------------------------------------------------------------------------------------------------

AEP_c25145_g1_i09 ----------------------------------------------------------------------------------------------------

AEP_c25145_g1_i04 -----------------TAGTAAAATCTGTAACGTCTGCGACAAAAGAGATTAAACATTTGATAG-----------------------------------

AEP_HAEP_T-CDS_v02_11692 ----------------------------------------------------------------------------------------------------

AEP_c25145_g1_i07 ----------------------------------------------------------------------------------------------------

AEP_HAEP_T-CDS_v02_7716 ----------------TTAGTAAAATCTGTAACGTCTGCGACAAAAGAGATTAAACATTTGATAG-----------------------------------

Ho_CS_S040478c1g2_i08 TTCGATTTGATTAGATTTAGTAAAATCTGTAACGTCTGCGACAAAAGAGATTAAACATTTGATAG-----------------------------------

Ho_CS_S040478c1g2_i03 ----------------TTAGTAAAATCTGTAACGTCTGCGACAAAAGAGATTAAACATTTGATAG-----------------------------------

Ho_CR_R039447c0g1_i05 ----------------TTAGTAAAATCTGTAACGTCTGCGACAAAAGAGATTAAACATTTGATAG-----------------------------------

Ho_CR_R039447c0g1_i02 ----------------TTAGTAAAATCTGTAACGTCTGCGACAAAAGAGATTAAACATTTGATAG-----------------------------------

1510 1520 1530 1540 1550 1560 1570 1580 1590 1600

...|....|....|....|....|....|....|....|....|....|....|....|....|....|....|....|....|....|....|....|

**C25145_g1_i04 E R M P I G H T E E S L F T K S S S I E E L D A V L L**

Hm-105_lcl|Sc4wPfr_396.1 ttggtcatactaagagacagAGCGTATGCCAATTGGTCACACTGAAGAAAGTTTATTCAAGAAATCAAGTAACATAAAAGAGTTAGATGCTGTGTTGTTG

Hm-105_lcl|Sc4wPfr_1246 ----------------------------------------------------------------------------------------------------

AEP_c25145_g1_i09 ----------------------------------------------------------------------------------------------------

AEP_c25145_g1_i04 --------------------AGCGCATGCCAATTGGTCATACTGATGAAAGTTTATTTAAGAAAGCAAGTAATGTAGAAGAGTTAGATGCCATGTTGTTG

AEP_HAEP_T-CDS_v02_11692 ----------------------------------------------------------------------------------------------------

AEP_c25145_g1_i07 ----------------------------------------------------------------------------------------------------

AEP_HAEP_T-CDS_v02_7716 --------------------AGCGTATGCCAATTGGTCATACTGAAGAAAGTTTATTTACGAAAT-----------------------------------

Ho_CS_S040478c1g2_i08 --------------------AGCGTATGCCAATTGGTCATACTGAAGAAAGTTTATTTACGAAATCAAGCAGCATAGAAGAGTTAGATGCTGTTTTGTTG

Ho_CS_S040478c1g2_i03 --------------------AGCGTATGCCAATTGGTCATACTGAAGAAAGTTTATTTACGAAATCAAGCAGCATAGAAGAGTTAGATGCTGTTTTGTTG

Ho_CR_R039447c0g1_i05 --------------------AGCGTATGCCAATTGGTCATACTGAAGAAAGTTTATTTACGAAATCAAGCAGCATAGAAGAGTTAGATGCTGTTTTGTTG

Ho_CR_R039447c0g1_i02 --------------------AGCGTATGCCAATTGGTCATACTGAAGAAAGTTTATTTACGAAATCAAGCAGCATAGAAGAGTTAGATGCTGTTTTGTTG

1610 1620 1630 1640 1650 1660 1670 1680 1690 1700

....|....|....|....|....|....|....|....|....|....|....|....|....|....|....|....|....|....|....|....|

**C25145_g1_i04 Q C Q D E E M A S L F A S F T ***

Hm-105_lcl|Sc4wPfr_396.1 CAATGTCAGGATGAAGAAATGGCCTCAATTTTTGCAAGTTTTATTTAAATATTATTTAAATACTTTTAAATTAAAACTCCGGTTGCATCCATTCAAAATC

Hm-105_lcl|Sc4wPfr_1246 ----------------------------------------------------------------------------------------------------

AEP_c25145_g1_i09 ----------------------------------------------------------------------------------------------------

AEP_c25145_g1_i04 CAATGTCAGGATGATAAAATGACC----------------------------------------------------------------------------

AEP_HAEP_T-CDS_v02_11692 ----------------------------------------------------------------------------------------------------

AEP_c25145_g1_i07 ----------------------------------------------------------------------------------------------------

AEP_HAEP_T-CDS_v02_7716 ----------------------------------------------------------------------------------------------------

Ho_CS_S040478c1g2_i08 CAATGCCAGGATGAAGAAATGGCCTCACTTTTTATTAACTGGCTAGCAACAGCAGGTGGAAAGTATATATCTGATATGGTACGAAACATTTTGAATGTTT

Ho_CS_S040478c1g2_i03 CAATGCCAGGATGAAGAAATGGCCTCACTTTTTATTAACTGGCTAGCAACAGCAGGTGGAAAGTATATATCTGATATGGTACGAAACATTTTGAATGTTT

Ho_CR_R039447c0g1_i05 CAATGCCAGGATGAAGAAATGGCCTCACTTTTTGCAAGTTTTACTTAAATATTATTGAAAAACTTTTTAATAAAAACTCCTGTTGCATCCATTGAAAAAT

Ho_CR_R039447c0g1_i02 CAATGCCAGGATGAAGAAATGGCCTCAATTATTGGAAGTTTTACTTAAATATTATTGAAAACATTTTTTATAAAAACTCCGGTTGCAATCATTGAAAAAT

### S2 Fig. Alignment of the *Hydra* genomic and transcriptomic *ms-c25145* sequences.

Alignment of the *ms_c25145* sequences identified in the *AEP* transcriptomes with three isoforms in the *AEP1* strain (*c25145_g1_i04*, *c25145_g1_i07*, *c25145_g1_i09)* and two in the uncharacterized *AEP* strain *HAEP* (HAEP_T-CDS_v02_11692 and HAEP_T-CDS_v02_7716). The *ms_c25145* sequences were also identified in *H. oligactis* transcriptomes with two isoforms in *Ho_CS* (S0404778c1g2_i08, S0404778c1g2_i06) and two in *Ho_CR* (R039447c0g1_i05, R039447c0g1_i02). These transcripts were detected in the *Hm-105* genome (lcl|Sc4wPfr_396.1, lcl|Sc4wPfr_1246). For details, see **S2 Table**. Primer sequences used for *ms_c25145* amplification are underlined and highlighted in grey. The microsatellite region, highlighted in yellow, is intronic (first intron). Putative intronic sequences are written lowercase. The *c25145_g1_i07* putative protein product (boxed) shows similarities with putative proteins from bilaterians (see **S4 Fig**).
