# Supplementary material for: The polymorphism of Hydra microsatellite sequences provides strain-specific signatures
Source: PLoS One. 2020 Sep 28;15(9):e0230547. doi: 10.1371/journal.pone.0230547 (PMC7521734; doi:10.1371/journal.pone.0230547)
Supplement: S3 Fig — (DOCX) [file pone.0230547.s005.docx]

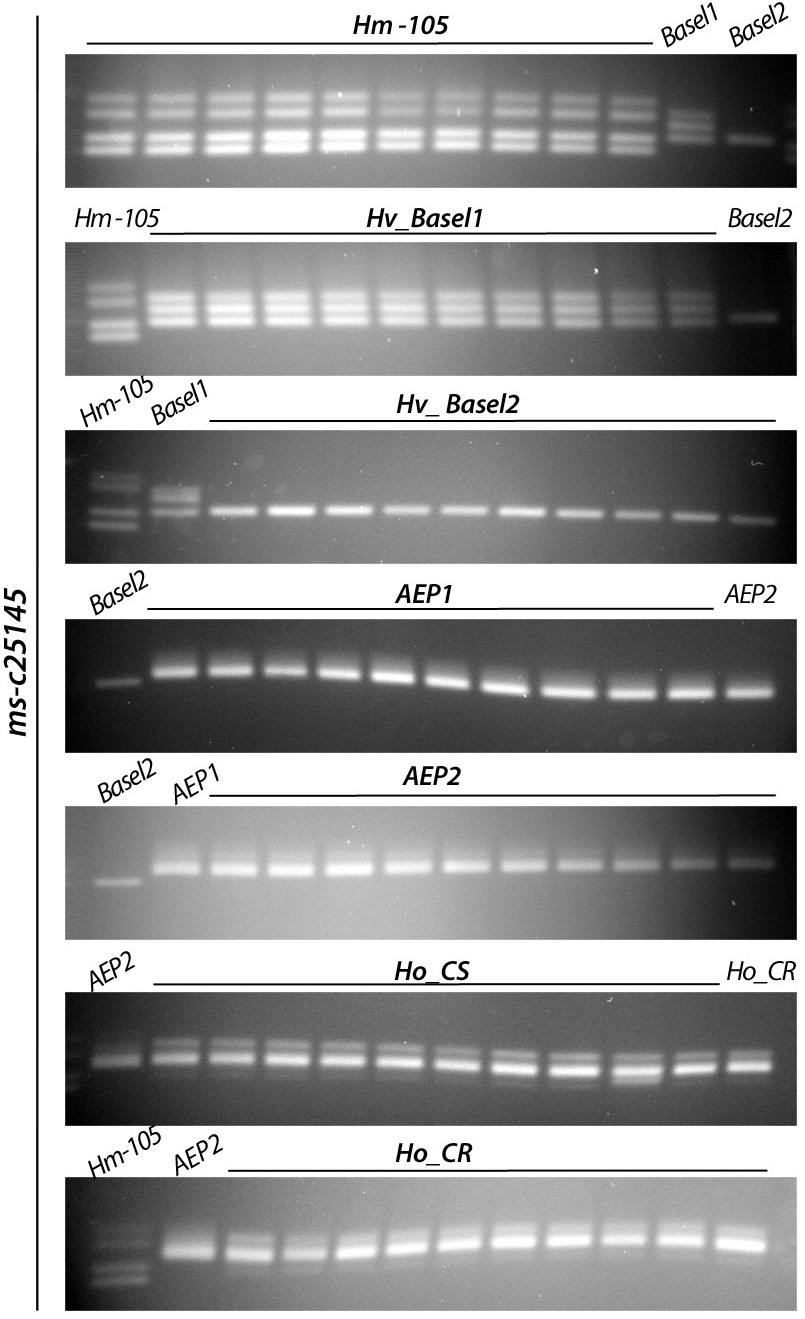


### S3 Fig. Reproducibility of the amplified patterns corresponding to the *ms-c25145* region.

For each strain, 10 animals were macerated individually and the *ms-c25145* region amplified.
