# Supplementary material for: The polymorphism of Hydra microsatellite sequences provides strain-specific signatures
Source: PLoS One. 2020 Sep 28;15(9):e0230547. doi: 10.1371/journal.pone.0230547 (PMC7521734; doi:10.1371/journal.pone.0230547)
Supplement: S4 Fig — (DOCX) [file pone.0230547.s006.docx]

#### Bilaterian sequences related to the orphan Hydra c25145 gene product

10 20 30 40 50 60 70 80 90 100

....|....|....|....|....|....|....|....|....|....|....|....|....|....|....|....|....|....|....|....|

**Hydra C25145_g1** --------MFYIVHFLNDNTVEYVPKEWLNGNSECMWPKC-SMTSLKGMRRKRQIPNKDWERYKIRILST--ADCEERALEKLKISEETSDLVSEYEGNS

Crassostrea gigas --------MFAVVHFVDDESVECVPKSWLEGQY-CYWP---HLNAKKKIQKMSLPDKDTWKKYKYRKIGQ-DYEDYDTARKNLKKAEETSNLESEEESRK

Danio rerio --------MFHIVEFIESSEVEVVPSSWVQNGA-CAWPSYKSMAKIHKAVTLQDSPNQSWATFRVRIIYT--TDSYEEARLKLPQATVMSDLQTDEDDDR

Myripristis murdjan --------MFKIVEFLETNEVELVPGAWVKDNV-CLWPAL-RGKALETAIKQQVSPGPDWMTWNIRVMFT--TDNYQEGRQKVREAERRSDLQSDAEDCS

Sinocyclocheilus rhinocerous MSPCEFAGMFHIVSFVETNEVEVVPSSWVHDEQ-CVWPNL-KGESLTKAVKLAMKPRKDWKKFRVKLLYT--TDNYEDARKKLPEAEVFSDIQSDAEGGV

Ixodes scapularis XRATPDDKKFGVVKFVGDNTVAVIHLNWVDGAD-CFWPTA-THKNLGALTLEGAQPQPDWKKSRFASLGW--YDTYQKATSKLPTAELTSDLCSDVEMGR

Nylanderia fulva -------MSWRVVHFIKDNTVEAGPALWVKDINGCFWPPC-SGLKLKNLIKNCVPPGHDWDLHQSRLIGE-LYGDLNVAKNKAAQAEETSDLASENEGSK

Photinus pyralis ----MNSKTWTVVQFLDDLTVEAIPSTWIQGNE-CHWPSF-SMEKLHNAIRKSEPLNTCWPTHKIKIFRNATYGDYLKARNKARIAENTSDINTEPEDVE

Sipha flava --------MWSIISFDNENAVEDVPSHWMKNNT-CVWP---KKDVKKHIQRRTNPNKFDFNYFKSRILKK-GIETLHETREKVKLAEDTSDLSN-IENSK

110 120 130 140 150 160 170 180 190 200

....|....|....|....|....|....|....|....|....|....|....|....|....|....|....|....|....|....|....|....|

**Hydra C25145_g1** CHRKTTSKRLSPSLFASQIHGN-------------MSSEDDSDFNMPTSLQQAVISTSTPGSQPLLPIPHHANVFREMQHQGKSSQSFVSLLNDPDEINL

Crassostrea gigas RKLPARLISESDNDTDIESDIEGKTLPSLPKNTCSQKEKKRTTPVKKMPTLPSLDSSPLSPSPPASSVPVKKQNLATHSVKRQLLTSTTSIRRSPRSKST

Danio rerio PSYTKRKNSDSDEEILSGRKRLG--------------KKGRMEDLTEIDDAPHIPSPPMTTAESFRTPARCTDTAHPSTPRNIYSPCSTAVGNQERTGCN

Myripristis murdjan GRKARRKTPSTRLQDGAHLTDS-------------EDEAGPQQRNNGLPSAPQVSPPTYATLHPPMISHQSSASQSQNEMRHEVCQSPSSTYWNADQDHR

Sinocyclocheilus rhinocerous KKPRRIMKSFRLQNFEVFNYSD-------------DDDDDDYGQNLRALEPPPHVQPPTFQSVSPLCQPASQPPASQPEGSKPSFYNPASQPPLTHQPSL

Ixodes scapularis GRRKKAKRILYSETES---------------------------------EGEET----------------------------------------------

Nylanderia fulva RKIKRKRFTSGSDSESRPITVN-------------TFTKRKSNIHKDSEESEES----------------------------------------------

Photinus pyralis VKRKRIQKILSSSEESIDDTIL-------------PPPPSISKYKAKKKTSTSTSTFKEVHITPGNDLGNGV----------------------------

Sipha flava WKKKTTAINLEP----------------------------------------------------------------------------------------

210 220 230 240 250 260 270 280 290 300

....|....|....|....|....|....|....|....|....|....|....|....|....|....|....|....|....|....|....|....|

**Hydra C25145_g1** PSLYQEFLPIPYTKNSTNT------------------LTPITQSIEYRLSSIENLLKDLVKSVTSATKEIKHLIERMPIGHTEESLFTKSSSIEELDAVL

Crassostrea gigas LSASSAGTATPPRSTPSVSIQLPVAASDVRTSATKTLGESISDLLKQVISKLDKQQQDITAIKNHLISAVDVDLDTIESLLPSGNRLNTSAEVEEFQDSL

Danio rerio SSCLS-------------------------------LLTEVIKAQEVMKQQLDVILKKLHKQNSTLQCEDIPEPSTFDLPLSNLLDLEKLECQIKEQP--

Myripristis murdjan TSVQWHHHFE----------------------------SSQGHQKQPYAWTMQNSSKSGSYSEMCARTDAGHPSTLMDGTPQDSTQIQVPLVPQQNNTYH

Sinocyclocheilus rhinocerous YQAASQLPSNRPVSDETSLNKPAGRPSLTHQPSLTHQPSHYQPASQPSLTHQPSLYEPASHPPLTHQPSLTHQPSHYQLASQPPVILQSSLFQSAIQPSS

Ixodes scapularis ----------------------------------------------------------------------------------------------------

Nylanderia fulva -----------------------------------------DESDTDNEINVPILKKTTKENSMTAASKIWLIFNLIQMKPTKDR---------------

Photinus pyralis ----------------------------------------HEATDNSFFENIENMHDDINDNYLEAENNNTCKNCRCKDCLEKDRALDRTNKQLMQ----

Sipha flava ------------------------------------------------PQYIEKLHKNKRNDDLDARKILQ-----------------------------

310 320 330 340 350 360 370 380 390 400

....|....|....|....|....|....|....|....|....|....|....|....|....|....|....|....|....|....|....|....|

**Hydra C25145_g1** LQCQDEEMASLFASFT------------------------------------------------------------------------------------

Crassostrea gigas DDDSKKKLINAMASLQGGEHAGEICRAVMRSIMTNNCMSQFSGTGQKGKIAFIGTPLYKIILSAVRKASKKTIPFETIKREVLDVLRFAPHLPGGINYAK

Danio rerio ---EQMKKLVAYFGIIGGFSTKEAVWRILGKLLANSLAKQINWSGANQKVAFRTLTLRTVVVNAVRTNGHTKSATDKEVEKYITRWLQLAPDRDGGRKER

Myripristis murdjan SNPWSQPGPTPAWTPRAALETITSVHP-------------------------------------------------------------------------

Sinocyclocheilus rhinocerous DNSPTLCVSQSHSKEPADSESVFRRNQQFQSMLYNCATLKLKNDGAQFRHRLPVKDMESLLCLEAELKNLDTKSELLPFVCRARNTTN------------

Ixodes scapularis ----------------------------------------------------------------------------------------------------

Nylanderia fulva ----------------------------------------------------------------------------------------------------

Photinus pyralis ----------------------------------------------------------------------------------------------------

Sipha flava ----------------------------------------------------------------------------------------------------

410

....|....|....|....

**Hydra C25145_g1** -------------------

Crassostrea gigas KKRGKKSNPKEGEFPPDSE

Danio rerio QKTNV--------------

Myripristis murdjan -------------------

Sinocyclocheilus rhinocerous -------------------

Ixodes scapularis -------------------

Nylanderia fulva -------------------

Photinus pyralis -------------------

Sipha flava -------------------

### S4 Fig. Alignment of the putative *Hydra* c25145 protein with related gene products identified in bilaterian species.

Protein from *Crassostrea gigas* (XM_020063127), *Danio rerio* (XM_001344599.3), *Ixodes scapularis* (XM_003920497.1), *Myripristis murdjan* (XM_030072039.1), *Nylanderia fulva* (XM_029320748), *Photinus pyralis* (XM_031502844.1), *Sinocyclocheilus rhinocerous* (XM_016559820.1) and *Sipha flava* (XM_025570128.1) were aligned with the deduced protein product encoded by the transcript *c25145_g1_i07* from *Hydra AEP* (HydrATLAS.unige.ch). Amino acids conservation is indicated by grey and black backgrounds (50% similar and identical, respectively).
