# Supplementary material for: The polymorphism of Hydra microsatellite sequences provides strain-specific signatures
Source: PLoS One. 2020 Sep 28;15(9):e0230547. doi: 10.1371/journal.pone.0230547 (PMC7521734; doi:10.1371/journal.pone.0230547)
Supplement: S5 Fig — (DOCX) [file pone.0230547.s007.docx]

#### Alignment of Hydra ms-AIP sequences

#### >AIP_HyAEP c8134_g1_i01, 333 amino acid long

MEEWLSLPDGVNKIILAAGHGDIPSFPDGAKVLFHYRAFSVNDDGEQKILDDSRADNAPFELLLGKKFKLEIWEALIKTMRINEIAEFHCDIKHVSTYPVVSKSLRDMKKKANKHDHDHNHEPGHQCGFAALSQGLGYSDLDEYYKDPKPLKFQIELLKVDLPGEYEQDVWSLTPEQQLQQIPVWKEEGNTYFRKGELDSASYKYSQALGCLEKLILREKPGSEEWIALDNMKIPLLLNFSQCMIAKKEYYKAIEHLTTVIEKDKNNVKALFRRAQAYHAVFSLLESRQDYEAVKKLDSSLLNTVEIELKKISLDEKNKEKEDREIFKKAFSS

10 20 30 40 50 60 70 80 90 100

....|....|....|....|....|....|....|....|....|....|....|....|....|....|....|....|....|....|....|....|

Hm-105_lcl|Sc4wPfr_417.3 GGATACTAACCTTTTCCCCAGCGCATTTAATTTTAAAAACAATTCGTCGAGACAGCGTTTTCAAGATATAACAA-CTTCCAGCAAATTTTATTCTTTATT

AEP_c8134_g1_i1 -----------------------------------------------------------------------------------------TATTCTTTATT

AEP_HAEP_T-CDS_v02_12771 ------------------------------TTTT-AAAACAATTCGCCGAGACAGCGTTTTCAAGATATAACAAACTTCCAGCAAATTTTATTCTTTATT

AEP_HAEP_T-CDS_v02_16467 ----------------------------------------------------------------------------------------------------

110 120 130 140 150 160 170 180 190 200

....|....|....|....|....|....|....|....|....|....|....|....|....|....|....|....|....|....|....|....|

Hm-105_lcl|Sc4wPfr_417.3 TATTGTTCATCAAAAAAAATAAAAAATAAAAATATATATATATATATATATATATATATATATATATATATATATATATATATATATCTATAAATATAAA

AEP_c8134_g1_i1 TATTGTTCATCAAAAAAAAA----------------------------------TATATATGTATATATATATTTATTTATATATGTTAAAAAGTTTAAA

AEP_HAEP_T-CDS_v02_12771 TATTGTTCATCAAAA---------------------------TATATATATATATATATATGTATATATATATTTATTTATATATGTTAAAAAACTTAAA

AEP_HAEP_T-CDS_v02_16467 ----------------------------------------------------------------------------------------------------

210 220 230 240 250 260 270 280 290 300

....|....|....|....|....|....|....|....|....|....|....|....|....|....|....|....|....|....|....|....|

**AIP_AEP** M E E W L S L P D

Hm-105_lcl|Sc4wPfr_417.3 AAACAAAGAAAAAAAGTAAATAAATACTTCGAATAACACATTGCTAGTTACATATTTATAAAAATTGGTTAGAATGGAAGAATGGTTATCATTACCAGAT

AEP_c8134_g1_i1 AATTTTTGCAAAAAGGTAAAAAGATACTTCGGATAACACATTGCTAATTACATATTTATAAAAGTTGGTTAGAATGGAAGAGTGGTTATCATTACCAGAT

AEP_HAEP_T-CDS_v02_12771 AATTTTTGCAAAAAGGTAAAAAGATACTTCGAATAACACATTGCTAATTACATATTTATAAAAGTTGGTTAGAATGGAAGAGTGGTTATCATTACCAGAT

AEP_HAEP_T-CDS_v02_16467 ----------------------------------------------------------------------------------------------------

310 320 330 340 350 360 370 380 390 400

....|....|....|....|....|....|....|....|....|....|....|....|....|....|....|....|....|....|....|....|

**AIP_AEP** G V N K I I L A A G H G D I P S F P D G A K V L F H Y R A F S V N

Hm-105_lcl|Sc4wPfr_417.3 GGAGTTAATAAGATTATACTTGCTGCTGGTCATGGCGATGTTCCTAGCTTTCCAGATGGAGCAAAAGTTTTATTTCATTATCGAGCATTCAGTGTCAACG

AEP_c8134_g1_i1 GGAGTAAATAAGATTATACTTGCTGCTGGTCATGGCGATATTCCTAGCTTTCCAGATGGAGCGAAAGTTTTATTTCATTATCGAGCATTCAGTGTCAACG

AEP_HAEP_T-CDS_v02_12771 GGAGTAAATAAGATTATACTTGCTGCTGGTCATGGCGATATTCCTAGCTTTCCAGATGGAGCGAAAGTTTTATTTCATTATCGAGCATTCAGTGTCAACG

AEP_HAEP_T-CDS_v02_16467 ----------------------------------------------------------------------------------------------------

410 420 430 440 450 460 470 480 490 500

....|....|....|....|....|....|....|....|....|....|....|....|....|....|....|....|....|....|....|....|

**AIP_AEP** D D G E Q K I L D D S R A D N A P F E L L L G K K F K L E I W E A L

Hm-105_lcl|Sc4wPfr_417.3 ATGACGGGGAACAAAAGATTTTGGATGATTCAAGAGCTGATAATGCACCATTTGAGTTACTACTCGGTAAGAAGTTTAAATTGGAAATTTGGGAAGCATT

AEP_c8134_g1_i1 ATGATGGGGAACAAAAGATTTTAGATGATTCAAGAGCTGATAATGCACCATTTGAGTTACTACTCGGTAAGAAGTTTAAGTTGGAAATTTGGGAAGCATT

AEP_HAEP_T-CDS_v02_12771 ATGATGGGGAACAAAAGATTTTAGATGATTCAAGAGCTGATAATGCACCATTTGAGTTACTACTCGGTAAGAAGTTTAAGTTGGAAATTTGGGAAGCATT

AEP_HAEP_T-CDS_v02_16467 ----------------------------------------------------------------------------------------------------

510 520 530 540 550 560 570 580 590 600

....|....|....|....|....|....|....|....|....|....|....|....|....|....|....|....|....|....|....|....|

**AIP_AEP** I K T M R I N E I A E F H C D I K H V S T Y P V V S K S L R D M K

Hm-105_lcl|Sc4wPfr_417.3 AATTAAAACAATGAGAATAAATGAAATTGCTGAATTTCATTGTGATATAAAGCATGTTTCTACTTACCCTGTTGTTTCTAAAAGCTTAAGAGATATGAAG

AEP_c8134_g1_i1 AATTAAAACAATGAGAATAAATGAAATTGCCGAATTTCATTGTGATATAAAGCATGTTTCTACATACCCTGTTGTTTCTAAAAGTTTAAGAGACATGAAG

AEP_HAEP_T-CDS_v02_12771 AATTAAAACAATGAGAATAAATGAAATTGCCGAATTTCATTGTGATATAAAGCATGTTTCTACATACCCTGTTGTTTCTAAAAGTTTAAGAGACATGAAG

AEP_HAEP_T-CDS_v02_16467 ----------------------------------------------------------------------------------------------------

610 620 630 640 650 660 670 680 690 700

....|....|....|....|....|....|....|....|....|....|....|....|....|....|....|....|....|....|....|....|

**AIP_AEP** K K A N K H D H D H N H E P G H Q C G F A A L S Q G L G Y S D L D

Hm-105_lcl|Sc4wPfr_417.3 AAAAAAGCTAATAAACACGACCATGATCACAATCATGAACCAGGACACCAATGTGGGTTTGCAGCACTTTCACAAGGTTTAGGGTATTCTGATTTAGATG

AEP_c8134_g1_i1 AAAAAAGCCAACAAACACGACCATGATCACAATCATGAACCAGGACACCAATGTGGGTTTGCAGCACTTTCACAAGGTTTAGGGTATTCTGATTTAGATG

AEP_HAEP_T-CDS_v02_12771 AAAAAAGCCAACAAACACGACCATGATCACAATCATGAACCAGGACACCAATGTGGGTTTGCAGCACTTTCACAAGGTTTAGGGTATTCTGATTTAGATG

AEP_HAEP_T-CDS_v02_16467 ----------------------------------------------------------------------------------------------------

710 720 730 740 750 760 770 780 790 800

....|....|....|....|....|....|....|....|....|....|....|....|....|....|....|....|....|....|....|....|

AIP_AEP E Y Y K D P K P L K F Q I E L L K V D L P G E Y E Q D V W S L T P E

Hm-105_lcl|Sc4wPfr_417.3 AATACTACAAAGATCCAAAACCTTTAAAGTTTCAAATTGAATTGCTTAAAGTAGATCTTCCAGGCGAATATGAACAAGATGTTTGGTCTTTAACTCCTGA

AEP_c8134_g1_i1 AATACTACAAAGATCCAAAACCTTTAAAGTTCCAAATTGAATTGCTTAAAGTAGATCTACCAGGCGAATATGAACAAGATGTTTGGTCTTTGACTCCTGA

AEP_HAEP_T-CDS_v02_12771 AATACTACAAAGATCCAAAACCTTTAAAGTTCCAAATTGAATTGCTTAAAGTAGATCTACCAGGCGAATATGAACAAGATGTTTGGTCTTTGACTCCTGA

AEP_HAEP_T-CDS_v02_16467 ----------------------------------------------------------------------------------------------------

810 820 830 840 850 860 870 880 890 900

....|....|....|....|....|....|....|....|....|....|....|....|....|....|....|....|....|....|....|....|

**AIP_AEP** Q Q L Q Q I P V W K E E G N T Y F R K G E L D S A S Y K Y S Q A L

Hm-105_lcl|Sc4wPfr_417.3 ACAACAACTTCAACAAATACCTGTTTGGAAGGAAGAAGGAAACACATTTTTTCGGAAAGGTGAACTTGACAGTGCAAGCAATAAATATTCTCAAGCTCTT

AEP_c8134_g1_i1 ACAGCAACTTCAACAAATACCTGTTTGGAAGGAAGAAGGAAACACATATTTTCGGAAAGGTGAGCTTGACAGTGCTAGCTATAAATATTCTCAAGCTCTT

AEP_HAEP_T-CDS_v02_12771 ACAGCAACTTCAACAAATACCTGTTTGGAAGGAAGAAGGAAACACATATTTTCGGAAAGGTGAGCTTGACAGTGCTAGCTATAAATATTCTCA-------

AEP_HAEP_T-CDS_v02_16467 --------------------------------------------------------------------------CTAGCTATAAATATTCTCAAGCTCTT

910 920 930 940 950 960 970 980 990 1000

....|....|....|....|....|....|....|....|....|....|....|....|....|....|....|....|....|....|....|....|

**AIP_AEP** G C L E K L I L R E K P G S E E W I A L D N M K I P L L L N F S Q

Hm-105_lcl|Sc4wPfr_417.3 GGTTGTTTAGAAAAACTAATTTTAAGAGAAAAACCAGGTTCAGAAGAATGGATTGTGTTAGACAATATGAAAATTCCTTTACTTTTAAATTACTCTCAGT

AEP_c8134_g1_i1 GGTTGTTTGGAAAAGCTAATTTTAAGAGAAAAACCAGGTTCAGAAGAATGGATTGCGTTGGACAATATGAAAATTCCTTTACTTTTAAATTTCTCTCAGT

AEP_HAEP_T-CDS_v02_12771 ----------------------------------------------------------------------------------------------------

AEP_HAEP_T-CDS_v02_16467 GGTTGTTTGGAAAAGCTAATTTTAAGAGAAAAACCAGGTTCAGAAGAATGGATTGCGTTGGACAATATGAAAATTCCTTTACTTTTAAATTTCTCTCAGT

1010 1020 1030 1040 1050 1060 1070 1080 1090 1100

....|....|....|....|....|....|....|....|....|....|....|....|....|....|....|....|....|....|....|....|

**AIP_AEP** C M I A K K E Y Y K A I E H L T T V I E K D K N N V K A L F R R A Q

Hm-105_lcl|Sc4wPfr_417.3 GTATGATTGCCAAAAAAGAATATTATAAAGCAATTGAACACTTAACTACTGTTATTGAGAAAGATAAAAATAACGTCAAGGCTCTCTTCAGAAGAGCACA

AEP_c8134_g1_i1 GTATGATTGCCAAAAAAGAATATTATAAAGCAATTGAACACTTAACTACTGTTATTGAGAAAGATAAAAATAATGTCAAGGCTCTCTTCAGAAGAGCACA

AEP_HAEP_T-CDS_v02_12771 ----------------------------------------------------------------------------------------------------

AEP_HAEP_T-CDS_v02_16467 GTATGATTGCCAAAAAAGAATATTATAAAGCAATTGAACACTTAACTACTGTTATTGAGAAAGATAAAAATAATGTCAAGGCTCTCTTCAGAAGAGCACA

1110 1120 1130 1140 1150 1160 1170 1180 1190 1200

....|....|....|....|....|....|....|....|....|....|....|....|....|....|....|....|....|....|....|....|

**AIP_AEP** A Y H A V F S L L E S R Q D Y E A V K K L D S S L L N T V E I E L

Hm-105_lcl|Sc4wPfr_417.3 AGCTTATCATGCTGTCTTCAATTTACGCGAATCACGTCAAGATTATGAGGCAGTGAAAAGACTTGATAGCTCATTATTAAATACTGTTGAAATTGAGTTG

AEP_c8134_g1_i1 AGCTTATCATGCTGTCTTCAGTTTACTCGAATCACGTCAAGATTATGAGGCAGTGAAAAAACTTGATAGCTCATTATTAAATACTGTTGAAATTGAGTTA

AEP_HAEP_T-CDS_v02_12771 ----------------------------------------------------------------------------------------------------

AEP_HAEP_T-CDS_v02_16467 AGCTTATCATGCTGTCTTCAGTTTACTCGAATCACGTCAAGATTATGAGGCAGTGAAAAAACTTGATAGCTCATTATTAAATACTGTTGAAATTGAGTTA

1210 1220 1230 1240 1250 1260 1270 1280 1290 1300

....|....|....|....|....|....|....|....|....|....|....|....|....|....|....|....|....|....|....|....|

**AIP_AEP** K K I S L D E K N K E K E D R E I F K K A F S S *

Hm-105_lcl|Sc4wPfr_417.3 AAAAAAATTTCTTTAGATGAAAAAAATAAAGAAAAAGAAGACAGAGAAATCTTTAAAAAAGCATTTTCAAGTTGAAATTTTTAATTTTTTTAAAAAACAG

AEP_c8134_g1_i1 AAAAAAATTTCTTTAGATGAAAAAAATAAAGAAAAAGAAGACAGAGAAATCTTTAAAAAAGCATTTTCAAGTTAAGAATTTTATTTAAAAAAAAAAAAAA

AEP_HAEP_T-CDS_v02_12771 ----------------------------------------------------------------------------------------------------

AEP_HAEP_T-CDS_v02_16467 AAAAAAATTTCTTTAGATGAAAAAAATAAAGAAAAAGAAGACAGAGAAATCTTTAAAAAAGCATTTTCAAGTTAAGAATTTTATTTAAAAAAAAAAAAAC

1310 1320 1330 1340 1350 1360 1370 1380 1390 1400

....|....|....|....|....|....|....|....|....|....|....|....|....|....|....|....|....|....|....|....|

Hm-105_lcl|Sc4wPfr_417.3 TATCAGCCAATATTTAAAGTTATAATAAAATTTACATTTTAAACAGTATCAGCCAATATTTATAAAAAAAGTTATGTTTATAAATGTTTAGTTTTTATGT

AEP_c8134_g1_i1 CAGTATCAGCCAATATTTAAAGTTATAATAAAATTTACATTTTAAACAGTATCAGCAAATATTTATAATAAAAGTTATGTTTATAAATATTTAGTTTTTA

AEP_HAEP_T-CDS_v02_12771 ----------------------------------------------------------------------------------------------------

AEP_HAEP_T-CDS_v02_16467 AGTATCAGCCAATATTTAAAGTTATAATAAAATTTACATTTTAAACAGTATC------------------------------------------------

1410 1420 1430 1440 1450 1460 1470 1480 1490 1500

....|....|....|....|....|....|....|....|....|....|....|....|....|....|....|....|....|....|....|....|

Hm-105_lcl|Sc4wPfr_417.3 AAGCCTTAAATTATTTTAATAAATAGTATTCAATGTCCACTAGTATTAATTTTAATTTTTTTCTTTGATATATATGGTTAGAGTGGTTACTTAATTATTT

AEP_c8134_g1_i1 TGTAAGCCTTAAATTATTTTGATAAATAGTTAGTATTCAATGTCCACTAGTAATAATTTTAATTTTCCCCATTGATATATAGGATTACAGTGG-------

AEP_HAEP_T-CDS_v02_12771 ----------------------------------------------------------------------------------------------------

AEP_HAEP_T-CDS_v02_12771 ----------------------------------------------------------------------------------------------------

#### Expression profile of ms_AIP along the body axis, in the different Hydra stem cell populations and during apical and basal regeneration


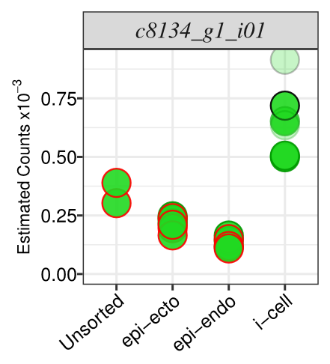

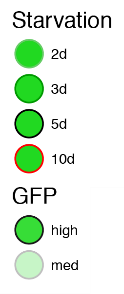

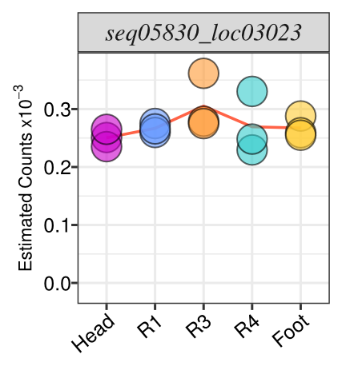

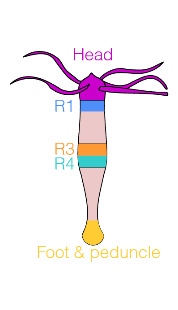


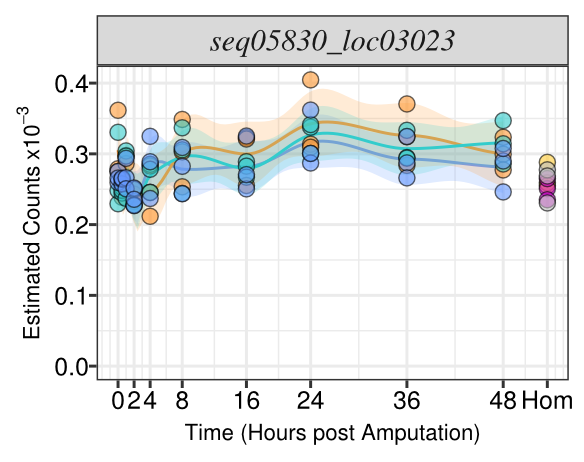

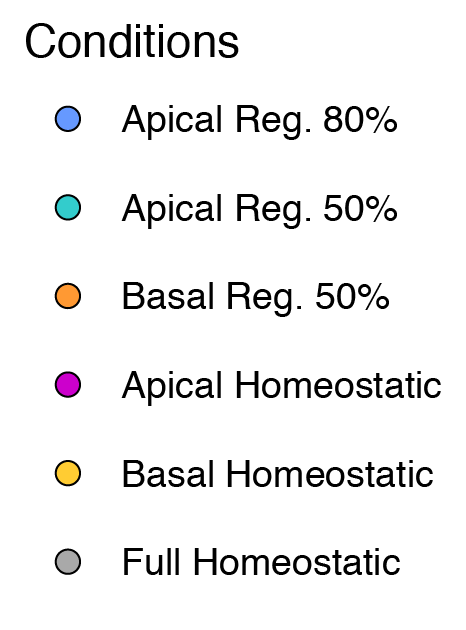


### S5 Fig. Alignment of the *ms-AIP Hydra* genomic and transcriptomic sequences and expression profiles.

**(A)** Alignment of the *ms-AIP* sequences identified in *AEP* transcriptomes (*c8134_g1_i1, HAEP_T-CDS_v02_12771, HAEP_T-CDS_v02_16467)* and *Hm-105* genome (*lcl|Sc4wPfr_417.3*, see **Table-S2**). The sequences coding for the predicted AIP protein are boxed. The microsatellite *ms-AIP* region, located in the 5’ untranslated region (5’UTR), is highlighted in yellow. The sequences of the primers used for *ms-AIP* amplification are underlined and highlighted in grey. **(B)** Expression levels of *AIP* in the three stem cell populations, either epithelial from the epidermis (epi-ecto) or epithelial from the gastrodermis (epi-endo), or interstitial (i-cell, left panel) ([HydrATLAS c8134_g1_i1](https://hydratlas.unige.ch/blast/get_sequences.cgi?hit_count=1&alignment_seq_1=c8134_g1_i01&database=full_database)). Expression levels of *AIP* along the body axis (right) or during regeneration (bottom panel) as depicted on the schemes (see [HydrATLAS seq05830_loc03023](https://hydratlas.unige.ch/blast/get_sequences.cgi?alignment_seq_1=seq05830_loc03023%7CHydra+vulgaris+Jussy&hit_count=22&database=db%2FHv_Jussy_transcriptome.fasta)).
