# Supplementary material for: The polymorphism of Hydra microsatellite sequences provides strain-specific signatures
Source: PLoS One. 2020 Sep 28;15(9):e0230547. doi: 10.1371/journal.pone.0230547 (PMC7521734; doi:10.1371/journal.pone.0230547)
Supplement: S6 Fig — (DOCX) [file pone.0230547.s008.docx]

#### Hydra ms-DMTF1

10 20 30 40 50 60 70 80 90 100

....|....|....|....|....|....|....|....|....|....|....|....|....|....|....|....|....|....|....|....|

**DMTF1_AEP M A N F**

c21737_g1_i04 ----------------------------------------------------------------------------------------------------

HAEP_T-CDS_v02_6413 GTAGTACGCGGGGACGGAAAAAAACACATACTGAAACTTTTTAGTCCCTGTGTAATAAGTTAATATACAAATAAAATAATATATAAAATGGCTAATTTTG

lcl|Sc4wPfr_307 TATTTTATAAAAGTGCATTTGTTTATCTAACATAAAATTTTTTTTTTTTTTTTTTTTAGTTAATATACAAACAAAACAATATAGAAAATGGCTAATATTG

110 120 130 140 150 160 170 180 190 200

....|....|....|....|....|....|....|....|....|....|....|....|....|....|....|....|....|....|....|....|

**DMTF1_AEP E N R Q K N T R T R K R R S N S D S E L V L A N Y S E K C F S A S S**

c21737_g1_i04 ----------------------------------------------------------------------------------------------------

HAEP_T-CDS_v02_6413 AAAACAGACAAAAAAATACACGTACTAGAAAGCGAAGATCAAATAGTGATAGTGAATTGGTGCTAGCCAACTATTCAGAAAAGTGTTTTTCAGCATCATC

lcl|Sc4wPfr_307 AAAACAGACAAAAAAATACACGTACTAGAAAGCGAAGATCAAATAGTGATAGTGAATTGGTGCTAGCCAACTATTCAGAAAAGTGTTTTTCAGCATCATC

210 220 230 240 250 260 270 280 290 300

....|....|....|....|....|....|....|....|....|....|....|....|....|....|....|....|....|....|....|....|

**DMTF1_AEP P D Q K Y F F N P S I G I N S Y A I D D I D I S A**

c21737_g1_i04 ----------------------------------------------------------------------------------------------------

HAEP_T-CDS_v02_6413 TCCTGATCAAAAATATTTCTTTAATCCTTCTATTGGAATTAATTCATATGCAATTGATGACATTGACATTTCTGCA------------------------

lcl|Sc4wPfr_307 TCCTGATCAAAAATATTTCTTTAATCCTTCTATTGGAATCAATTCGTATGCAATTGATGACATTGACATTTCTGCAGgtaaaattaagcaatttttattt

lcl|sc4wpfr_307 acaagcaaattgtaaataaatcatccaaattatatcttaactaggctatatattttatcgtaactttaacttatcatgtttaaattagcttaaaagtgtt 400

lcl|sc4wpfr_307 tgttgtgtttttattttaattttaaattcaattttaaaagtttattttatcaaattttgataacattcccaatgttatgtgttagtttaagttaattttt 500

lcl|sc4wpfr_307 ctttaaacaaagcatataagtttatatctttgttgcttatacagtgggatggatgtgtcacaaatttattatcttctcttataaaaatataattggtttt 600

lcl|sc4wpfr_307 agagttgagttagatttattgaaatcgtaaaattaatattagttagctatttgtgtagttgatgtgtttctaaatatccgcacaaataattttttttaaa 700

lcl|sc4wpfr_307 catgttaagaaaataagaataaaagaatattggaacttcnnnnnnnnnnnnnnnnnatacatatatatatatatatatatatatatatatatatatatat 800

lcl|sc4wpfr_307 atatatatatatatatatatatatatatatatatatatatatatatatatatatatattaattgtacaaagttgcttaaaaaagtaattgttgttttgat 900

lcl|sc4wpfr_307 ttaataagtaatgagttaattatgttgaaacttttcaaagaacttgagttgcttatcatgttgcacttgttttttttcaaaaataattaatgattctatt 1000

lcl|sc4wpfr_307 agtactggatgtttcccacaaaaattaaaactagctatgataaaacctgttaaatatcaaattgagaaaaatcttgatcataatgaaattattctgatta 1100

lcl|sc4wpfr_307 atcagtttagcatcaaaaagaagtgcaatgcagtacatactaatttaaaaattctcaatatgacaacggactatagaaaccaaaataaaatggttgcatt 1200

lcl|sc4wpfr_307 cattttacttgatataaaaaaagcttttgatacctgagaccattatttacaaaaagctcaaaaaacattgttgccagtgaaaaaaatttaaaacagatat 1300

lcl|sc4wpfr_307 cttcatatttataacatatattaacaaacagaaggtagtcagttgttattggaagttctacatcaaaagaaagagtagtaaagttaggtgttggtcaaga 1400

lcl|sc4wpfr_307 aacatgtatgcccattacttttcaaatttatatttatgatttaccagatgcaacagacttagacactatttgttttgctgttgatacaacaatgatggca 1500

lcl|sc4wpfr_307 attgtttacactattaaaaagttcaggaaatatgttgcaggaaattagaaaaaattaataattggtttttaataaacagatcacttcatcctgaaaaaac 1600

lcl|sc4wpfr_307 aaaaattaacgatttatggtaacgcaaaaaatatagaaatgttacaaaagcttaaggttacccaaagaaatttataaagaaaaaagtgttagcgttcttg 1700

lcl|sc4wpfr_307 gtattctttgggacgacaaaatgaaatggcatcgccaaaaaccatatagtagaaacatgaacaaagataagtaaaggcatctacctacttaaaaagttca 1800

lcl|sc4wpfr_307 ataaactattgcctagtaaatctaaagttgttttatacaatgctttaattagaagccatttaatgtatggtattgatatttaaaattgaaacagtaaatc 1900

lcl|sc4wpfr_307 aaaaaaaattatgcaactcaaatgtttacaaagctcctcagattattggtcctaacaaggtacacacaagaccaatcctaaaaaaattcgttattgttag 2000

lcl|sc4wpfr_307 atcaagaggattaaatggctattagaatccaaacatggaagatatgtgttggatcagcaccatcatctttaaataatgattttatctgggttaacattga 2100

lcl|sc4wpfr_307 aaaatctttgagagttaacagaagaatagctcttcctaactttaagagttgtgtgttggcaaatcaagcccctaacaaaattggaaatgaagctaattga 2200

lcl|sc4wpfr_307 ttgaaagaaaaaacctttgtgctcttaatatggcttcaaaatgtcacaatatctgttgtgctgaatgttaattgtaaaattttgtttgatttttttaaaa 2300

lcl|sc4wpfr_307 caatatatcaaatgtaaaaattaggaaataaagaagacacctttcattaagtattataccctttcagttttcaagttaaatttacctttttattgtaaat 2400

lcl|sc4wpfr_307 ttatataatactgaagaaattatttatatatatatatatatatatatatatatatatatatatatatatatatatatatatatatatatatatatatata 2500

lcl|sc4wpfr_307 tatatattattttatttttatttcaattcacctcctcaatgccgagaaggccactacagacgaggaggctacttatttgtgattataagcctctctcaac 2600

lcl|sc4wpfr_307 tctataactccgaaacatgaaccttgatgaataaggctgctgcgccgagaaacggtttgagtgcggtactaccagggacgtggtggaaatcaaactcgga 2700

lcl|sc4wpfr_307 accactcgtttatgaagcgagcgctctaccactacaccactaccgcatataaggagaacatgcaagggaaaataaggatataaggagaacatgtaaggaa 2800

lcl|sc4wpfr_307 aaagactgatgcaaggggaacaatgcaacatgcacacatatatatacaggagctattctaggaaaaaaatatgggcggcagtacccatgtatatatatgt 2900

lcl|sc4wpfr_307 atatatatatagaatagcccctgatatataaaggccttggcgggaagcgagagctttagctcttgcttcccgttgacactcccctaaattggtttactga 3000

3010 3020 3030 3040 3050 3060 3070 3080 3090 3100

....|....|....|....|....|....|....|....|....|....|....|....|....|....|....|....|....|....|....|....|

**DMTF1_AEP D N P F F N A R F T M D P A Q M F M S V K**

c21737_g1_i04 ----------------------------------------------------------------------------------------------------

HAEP_T-CDS_v02_6413 ---------------------------GATAATCCATTCTTCAATGCTCGTTTTACAATGGATCCTGCACAGATGTTTATGTCTGTTAAA----------

lcl|Sc4wPfr_307 agcaattaNNNNNNNNNNNNNNNNNNNNNNNNNNNNNNNNNNNNNNNNNNNNNNNNNNNNNNNNNNNNNNNNNNNNNNNNNNNNNNNNNNNNNNNNNNNN

lcl|sc4wpfr_307 nnnnnnnnnnnntacaccatacatatgaggcgggatgggatcactaggatatattttttcgtattcctcttttgaaaaacagtttcttttatttagtttt 3200

lcl|sc4wpfr_307 gaaagacatctacgaattttagctgcgtaatttgcagtagggtcttcacttactatttttgttgggcctatttgttcgcgaattttttccaaggcttttg 3300

lcl|sc4wpfr_307 aatgttcgattcgtacaaaacctgtccctttgtcgaatggatatatgtcaacgtttttttcctcttttatttctctataagctttccattgttcttttgt 3400

lcl|sc4wpfr_307 aagattattatttaatttttttccggtttttagttctcttaaaacatttttccttaggtcttgggcattttcaattttattgttgtattctaattttaac 3500

lcl|sc4wpfr_307 gcagaggattccgtggctgtaataatatccatatatggtatcgactttaaagatggtacaaagtgtggaccgaggtttagaaaattattatgacttactg 3600

lcl|sc4wpfr_307 gaatgtcctagttatttgggaacaaacaaaaacattaaaagtagaaaataaaaaatttgaaagaaaagttagggaagccctagaaatacaaaaaaacatg 3700

lcl|sc4wpfr_307 tgttctgcaaaaaatggcgggattaatctcgatgagggtcaatacgtaaaaactaagttttggacgccattttttaaatttcagcgaaaaagaagccatt 3800

lcl|sc4wpfr_307 caacagctgacgtcaatagcaacgttttaatttaaattataacgattttattaattactgtaacatttaacaagctgaagaagctggtatctaaaatcca 3900

lcl|sc4wpfr_307 gcgaaaatttctataataataaaaaaattataagtgttgagagaaatcgtatttttgatgttttaaaataatatataaatatactctcacacaagatgtt 4000

lcl|sc4wpfr_307 atcctttaaatcatatatatatatatatatattcctcaaattaaaagatcaggatgtgttttttatattatttatagatactataggcaattgtttatta 4100

4110 4120 4130 4140 4150 4160 4170 4180 4190 4200

....|....|....|....|....|....|....|....|....|....|....|....|....|....|....|....|....|....|....|....|

**DMTF1_AEP E H D A S L D I S V S S T Q I E N**

c21737_g1_i04 --------------------------------------------------------------------------------------------------

HAEP_T-CDS_v02_6413 -----------------------------------------------GAACATGATGCATCATTGGACATTAGTGTATCTTCAACACAAATTGAAAAT

lcl|Sc4wPfr_307 atatacactttgttaattaagaaataaaatttatatcttcaaatacaGAACATGATGCATCATTGAACATAAGTGTATCTTCAACACAAATTGAAAAT

4210 4220 4230 4240 4250 4260 4270 4280 4290 4300

....|....|....|....|....|....|....|....|....|....|....|....|....|....|....|....|....|....|....|....|

**DMTF1_AEP N P T E L C K N E H E Y M N S E Y L N N V N S Q Q**

c21737_g1_i04 ----------------------------------------------------------------------------------------------------

HAEP_T-CDS_v02_6413 AATCCAACAGAACTTTGCAAAAATGAACATGAATACATGAACTCTGAATACCTAAATAATGTTAATTCTCAGCAG-------------------------

lcl|Sc4wPfr_307 AATCCAACAGAACTTTGCAAAAACGAACATGAATTCATGAACTCTGAATACCTAAATAATGTTAATTCTCAGCAGgttggtattttatattgcattgata

lcl|sc4wpfr_307 aaaaattttataaactttaaaacaaaatctgactgggcctgaagatctgattacagcatgtgaagctatatattaattgatcccatgttcaattaattga 4400

lcl|sc4wpfr_307 gcattcagtatttttgaaattgttttattcactctaaatgtatgaaattaattttaaagcttttttgtggtctgttttagtataaattttaagagattca 4500

lcl|sc4wpfr_307 attgtcttttcactaaatactgttatatataaatttaattcataaattctattaaatgtagattttactctaaataaaccaatgtttactttttgtcatt 4600

lcl|sc4wpfr_307 ttattggtttagaaagtttattgaagttcaatttatgatattatacgatcttagtcaagtttaatttatatataagctatgattacatgattatatataa 4700

lcl|sc4wpfr_307 tatgcctaaaacacagttttaagcatagataaaagacacttgtcttttatggcccaaggaccatctcaaaagaaatcataaaaaaaatccctgtcagcca 4800

lcl|sc4wpfr_307 tataatagtagtaagaatgaggtttgatagaattgacttttatgagatttgatagaaatgacttttaagagattttatagaatttcttctctctactttt 4900

lcl|sc4wpfr_307 tgtgagctaatcccaaaatttgctttgtattttaaatggtctctataccaaataaactttctataagaattttatagaaaatctaaatataatgaaatct 5000

lcl|sc4wpfr_307 aagtatttataagttgcagaaaataaatgtgtacaaaatataatttttatacttgcataaataaaagttttatatttattaatttgttttaatatgttgc 5100

5110 5120 5130 5140 5150 5160 5170 5180 5190 5200

....|....|....|....|....|....|....|....|....|....|....|....|....|....|....|....|....|....|....|....|

**DMTF1_AEP D F Q T V S I N K D F E M**

c21737_g1_i04 ----------------------------------------------------------------------------------------------------

HAEP_T-CDS_v02_6413 ------------------------------GATTTTCAAACTGTTAGCATAAACAAAGACTTTGAAATG-------------------------------

lcl|Sc4wPfr_307 aacaatattctgaaatgtttttttcttcaGGATTTTCAAACTGTTAGCATAAGCAAAGACTTTGAAATGgtttcttatatacaaaattcaactgaaaaaa

#### Hydra ms-DMTF1

5210 5220 5230 5240 5250 5260 5270 5280 5290 5300

....|....|....|....|....|....|....|....|....|....|....|....|....|....|....|....|....|....|....|....|

**DMTF1_AEP I P Y I Q N S A E R K D C V N K M W S L M K E D K K N L I E**

c21737_g1_i04 ----------------------------------------------------------------------------------------------------

HAEP_T-CDS_v02_6413 -----------ATCCCTTATATTCAAAATTCAGCTGAAAGAAAAGATTGTGTTAATAAAATGTGGAGTCTGATGAAGGAAGATAAAAAAAATCTTATTGA

lcl|Sc4wPfr_307 aagactttgaaAATCCTTATATTCAAAATTCAGCTGAAAAAAAAGAGTGTGTTAACAAAATGTGGAGTCTAATGAAGGAAGATAAAAAAAATCTTATTGA

5310 5320 5330 5340 5350 5360 5370 5380 5390 5400

....|....|....|....|....|....|....|....|....|....|....|....|....|....|....|....|....|....|....|....|

**DMTF1_AEP K**

c21737_g1_i04 ----------------------------------------------------------------------------------------------------

HAEP_T-CDS_v02_6413 GAAAG-----------------------------------------------------------------------------------------------

lcl|Sc4wPfr_307 GAAAGgtaaaaacaaaaatttttatatctttaaatgagatgaaatccaatttatctaaagttatttatttatagataaatttaaatataaagtataaaaa

5410 5420 5430 5440 5450 5460 5470 5480 5490 5500

....|....|....|....|....|....|....|....|....|....|....|....|....|....|....|....|....|....|....|....|

**DMTF1_AEP G L E W K S G L W S P G E E**

c21737_g1_i04 -------------------------------------------------------------------------------------------------

HAEP_T-CDS_v02_6413 -------------------------------------------------------GTTTAGAATGGAAAAGTGGGTTATGGAGTCCTGGAGAGGAAA

lcl|Sc4wPfr_307 tgtatataaattattaagtggaaaattagtatcaaaatttaaaaatgtattttagGTTTAGAATGGAAAAGTGGGTTATGGAGTCCTGGAGAGGAAA

5510 5520 5530 5540 5550 5560 5570 5580 5590 5600

....|....|....|....|....|....|....|....|....|....|....|....|....|....|....|....|....|....|....|....|

**DMTF1_AEP I I L Q S N I T K Y C K**

c21737_g1_i04 ----------------------------------------------------------------------------------------------------

HAEP_T-CDS_v02_6413 TTATTTTACAATCAAACATCACCAAATATTGTAAA-----------------------------------------------------------------

lcl|Sc4wPfr_307 TCATTTTACAATCTAATATCACCAAATATTGTAAAgtgtgtcattttgagtttcaatagtgtttttgtttttgcacttttttaaataaaaagttctctat

lcl|sc4wpfr_307 aaatttattaaataatcaaactgctatatattactaatataacttgcatttctatatactaacatctcccactaacatcttacatctcccactaatgatc 5700

lcl|sc4wpfr_307 tcaatcttaactttcatttcttctcttttttctctacaaattttttttttaatttttttattttattttgtttttcaacaaaagttaaatgtatgaatgc 5800

lcl|sc4wpfr_307 tatgttttataaataactcagagtaaaatatatagcattacattgtgttttaaaaagtttttatcagcattatcactattgcctaaaaagaaaataatga 5900

lcl|sc4wpfr_307 tagattttaaacaaaggttgaatgtttaggaaaacgtttataaattaacggaaaaataggtaatctaggtattataaatacgtaattgatattataatta 6000

lcl|sc4wpfr_307 ttatgtaccaagttttatttgaaattaaaatttaccttataacttgttaaactaaaaattctttaagtgttaaactttggctcagatgaatatacacgtt 6100

lcl|sc4wpfr_307 actattttaattaaaacaagaaaggttatagtagataacctaaagttatagtagataacctaaagttatagtagataacctaaagttatagtagataatc 6200

lcl|sc4wpfr_307 taaagttatagtagataacctaaagttatagtagataacctaaagttatagtagataacctaaattaaaatttttgtgtttgtacaaaacatttaaaggc 6300

lcl|sc4wpfr_307 acgaaccagattctgttttttacttttgttgtttataataattttcatgctttgaaatcacctttttaaataactttttatttgaaaacttgcaatttcg 6400

lcl|sc4wpfr_307 gaaacaaaactttaattagtctagcagcaatttctttcgttacgggaactatatctttgtctattcaattataatttataataatagatactatttatag 6500

lcl|sc4wpfr_307 gaatagataattttttttgtttttaaagatagatttaatcgtaaaagtttttagaaaagcaaaatttaaaattgttttctatcattttgaaaatattttg 6600

lcl|sc4wpfr_307 accgcataaaaagtttagatttcaactttttttaattacatagtaattaaataatagtagcagtcagcgaaacaataaattgtttttaaaaaactaactt 6700

lcl|sc4wpfr_307 tacaagctttttttttttcaatacttaaatcagcgatcatggttcttttaaataaaaaattttaggactgccacctatgccacctaattcccacctgatt 6800

lcl|sc4wpfr_307 gtagcaattgttcaattcaactcttgattgcaccgcagttgatttattctttaaaatttgttaatcatttaaagaatatagtaaaataatttttttttaa 6900

lcl|sc4wpfr_307 aaggcccttctaatataaaaaagcttaaaacttatgtgagattcccgtatagcctctggggccagcaagcactggaatttcttgtatttgcaatttcgtt 7000

lcl|sc4wpfr_307 tgaaatatatgaaaataaaaatttcaagattgtgtaattacggttagtttattttgattgtattgtaaaattgttagattatttttctaatagtgagtat 7100

lcl|sc4wpfr_307 tttctaaaaaaatattgaaaataaagtgttttattacttattattattttacactcagttttgaaattattcatttaaagatttgtataagttaattttt 7200

lcl|sc4wpfr_307 gaaattttgaagcatcaaaaagtataaaagtttgagattcaagatttcatcaaataattctcttatttgtaaatataaatagtttttgtataatttctat 7300

lcl|sc4wpfr_307 aaaaatattttctgcattcattttacagtttttttatttcgaaatgttaaatcataaaaaagcatttgataggttttaaaagtattcaaaaaattttaat 7400

7410 7420 7430 7440 7450 7460 7470 7480 7490 7500

....|....|....|....|....|....|....|....|....|....|....|....|....|....|....|....|....|....|....|....|

**DMTF1_AEP K N G L S D A S I**

c21737_g1_i04 ----------------------------------------------------------------------------------------------------

HAEP_T-CDS_v02_6413 -------------------------------------------------------------------------AAAAATGGTTTATCTGATGCATCCATA

lcl|Sc4wPfr_307 ctattttattattcataaaagttaattaaaagtttattgaaaagaattatctaagttataaaaataactttagAAAAATGGTTTATCTGATGCATCCATA

7510 7520 7530 7540 7550 7560 7570 7580 7590 7600

....|....|....|....|....|....|....|....|....|....|....|....|....|....|....|....|....|....|....|....|

**DMTF1_AEP M I F S T P R E K R K E F Y R S I**

c21737_g1_i04 ----------------------------------------------------------------------------------------------------

HAEP_T-CDS_v02_6413 ATGATATTTTCAACTCCCAGAGAAAAGAGAAAGGAATTTTATCGATCTATAG------------------------------------------------

lcl|Sc4wPfr_307 ATGATATTTTCAACTCCCAGAGAAAAGAGAAAGGAATTTTATCGATCTATAGgttaataaatattaaaaaataaatcatcttttattactagtacaaata

7610 7620 7630 7640 7650 7660 7670 7680 7690 7700

....|....|....|....|....|....|....|....|....|....|....|....|....|....|....|....|....|....|....|....|

**DMTF1_AEP A I G L N R P L F T I Y R K V L**

c21737_g1_i04 ----------------------------------------------------------------------------------------------------

HAEP_T-CDS_v02_6413 -----------------------------------------------------CAATTGGTCTTAACCGACCATTATTCACTATTTATCGAAAAGTTTT

lcl|Sc4wPfr_307 tcacatataataaaatgatttttaaaattttagcattcaccttttaattttagCAATTGGTCTTAACCGACCATTATTCACTATTTATCGAAAAGTTTT

7710 7720 7730 7740 7750 7760 7770 7780 7790 7800

....|....|....|....|....|....|....|....|....|....|....|....|....|....|....|....|....|....|....|....|

**DMTF1_AEP R M C N Q K N Y V G K Y T Q V E V E K L A E L C R I H G N D W A T**

c21737_g1_i04 ----------------------------------------------------------------------------------------------------

HAEP_T-CDS_v02_6413 AAGAATGTGCAATCAAAAAAACTATGTTGGAAAGTATACACAGGTTGAAGTTGAAAAACTTGCTGAACTTTGTCGTATTCATGGGAATGATTGGGCTACA

lcl|Sc4wPfr_307 AAGAATGTGCAATGAAAAAAACTATGTTGGAAAGTATACACAGGTTGAAGTTGAAAAACTTGCTGAACTTTGTCGTATTCACGGGAATGATTGGGCTACA

7810 7820 7830 7840 7850 7860 7870 7880 7890 7900

....|....|....|....|....|....|....|....|....|....|....|....|....|....|....|....|....|....|....|....|

**DMTF1_AEP I G H H L G R S P G S V R D K A R L L K C H K K R**

c21737_g1_i04 ----------------------------------------------------------------------------------------------------

HAEP_T-CDS_v02_6413 ATAGGGCATCATCTTGGAAGAAGTCCTGGATCAGTTAGAGATAAAGCAAGACTTCTTAAATGTCATAAAAAGAGAG------------------------

lcl|Sc4wPfr_307 ATAGGGCATCATCTCGGAAGAAGTCCTGGATCAGTAAGAGATAAAGCAAGACTTCTTAAATGTCATAAAAAGAGAGgtttttaatttttttttacatttc

7910 7920 7930 7940 7950 7960 7970 7980 7990 8000

....|....|....|....|....|....|....|....|....|....|....|....|....|....|....|....|....|....|....|....|

**DMTF1_AEP G K W S E D E L K H**

c21737_g1_i04 ----------------------------------------------------------------------------------------------------

HAEP_T-CDS_v02_6413 ------------------------------------------------------------------------GTAAATGGAGTGAAGATGAGTTAAAACA

lcl|Sc4wPfr_307 aggggatgtgaattttttttatagcttatacctctaagtgttcaaaagtaaataaagctaaattgtttcagGTAAATGGAGTGAAGATGAGTTAAAACA

8010 8020 8030 8040 8050 8060 8070 8080 8090 8100

....|....|....|....|....|....|....|....|....|....|....|....|....|....|....|....|....|....|....|....|

**DMTF1_AEP L S D I V H A Q T N T K K G E S V T V G I N W A K V A E N I E T R**

c21737_g1_i04 ----------------------------------------------------------------------------------------------------

HAEP_T-CDS_v02_6413 TTTATCAGATATTGTTCATGCTCAAACAAACACTAAAAAAGGAGAAAGTGTAACTGTTGGAATAAACTGGGCAAAAGTAGCTGAAAATATTGAGACAAGA

lcl|Sc4wPfr_307 TTTATCAGACATTGTTCATGCTCAAACAAATACTAAAAAAGGAGAAAGTATAACTGGAGGAATAAACTGGGCAAAAGTAGCTGAAAATATTGAGACAAGA

#### Hydra ms-DMTF1

8110 8120 8130 8140 8150 8160 8170 8180 8190 8200

....|....|....|....|....|....|....|....|....|....|....|....|....|....|....|....|....|....|....|....|

**DMTF1_AEP T E K Q C R S K W**

c21737_g1_i04 ----------------------------------------------------------------------------------------------------

HAEP_T-CDS_v02_6413 ACTGAAAAACAATGTCGATCAAAATG--------------------------------------------------------------------------

lcl|Sc4wPfr_307 ACTGAAAAACAATGTCGATCAAAATGgtatttattaattaaaagtaaatgtttttctctgtttaataatttgtaattattgtaggcattgtctcatgctc

lcl|sc4wpfr_307 cagatgaagttgcctgttttaataacattaaaattttggtttctcaaggtcaatgcggtagtggtgtagtggtagagcgctcgcttcataaacgagtggc 8300

lcl|sc4wpfr_307 tccaagttcgatccccaccacgtccctggtagtaccgcgctcaactcatttctccgcgcagcggccttgttcgtcaaggttcgtgtttcggagttataga 8400

lcl|sc4wpfr_307 gttgagaaagggttataaccacaaataagtagcctcctcatctgtagtggccttcacggccttgaggaggtgaattaaaacaaaaaaaaaaacaaaaaaa 8500

lcl|sc4wpfr_307 aaatacctgaaaagaacattgagagatgttctttttaggtatttgccccctcctccccctccccatattgcttggtacctgaaattatggttttcaattt 8600

lcl|sc4wpfr_307 gtttcttttagatttgtctaataaagttgtctattagagtttgtgtttctgagagagaagcgtgacaaagaaagtgattaaaaaaattacgataaaaaaa 8700

lcl|sc4wpfr_307 atcttatattgttaaataattaactttgtaaaaaaattatttgtatatttattttttctaaatatttttctgaaaaggagttttgcatgttctacatata 8800

lcl|sc4wpfr_307 tattagtcaatgtagtagcactcctcctatttgttgatgtaaagagtaaaataataaaaattatcatttagaatgtttttattaaaaaaattaaaaaaac 8900

lcl|sc4wpfr_307 attcaagcctataaagtttttaggttttttaaataatgataaaatttaagtttcctcaattaaattaacttttacataaagtgtacttgacgtcgctttt 9000

lcl|sc4wpfr_307 aacgacaaagagttgttaagagttttaccacaaacagtaaattttgtgtgtgtgtgtgtgtgtgtgtgtatttagatatttagatattaagtatgttttt 9100

lcl|sc4wpfr_307 ttcttttttctttaaaaaaaataaagttaaataaaaaataataatgtactttaagtggaaataggttttctgttggttttttttaccttttttcacatac 9200

lcl|sc4wpfr_307 tttttactcctcaactattttttttaacttttttgaaagttttaaaaagagttataaaaatgctaaatttttttgattttcgagctgaaaatagtgaaat 9300

lcl|sc4wpfr_307 ggtaaaatttcaagtttttgcagttgcaatttttgggctaccaatgggaacttggcagtgcacaataaatgttgtgcttgctcagtttctattggatgtc 9400

lcl|sc4wpfr_307 tttaattcaggtttattacctaacactaggcagttaaatagtttttcgtttagagggctaagtactacacatgttaagtcaagttcaccttttttctttt 9500

lcl|sc4wpfr_307 aatcaataatatacttaaaatattaagcattaaagactaacttttaaattaaaattctttaatatttatatactttcaatttttttttaatgatgacata 9600

lcl|sc4wpfr_307 aacctttctgttataataattaacaagtgagctgtaggagataattagcagctgtaggagataattaacagctgtaggagataatgaggagagatcactc 9700

lcl|sc4wpfr_307 tcgagatcagctctccaagtgatcaaaatgtgatgactttttattgagactgaagtatatagttgtgctgtcagcaaataaggaagcgaatacggaattt 9800

lcl|sc4wpfr_307 ataagaaaggttgtcagaaagatcattaatgtagatgaaaatgatacaggattttacattacttttttttaaataatgttaatttattaaatttattctt 9900

lcl|sc4wpfr_307 ttcttcactagagtagtagtaaatatttttatttcaaagacttttctaataatagtaagaaggctcattgacctataaaatttctaaaaataaagtcagt 10000

lcl|sc4wpfr_307 gtttgaacaataagtctgtctgaacaatctccagaatttttaagtcacatattttacagtagggaaaaaaattatttgttaaatattttagaggttaata 10100

lcl|sc4wpfr_307 aaagagagtattaaattgtggacatttatgtgaacattgtgctgtagacatttgtaaaagtgtgacagtggagaacacatttgtaagagtgtaacagtgg 10200

lcl|sc4wpfr_307 ggaaaaatttgtaagattgtgacagtgaaaaacacatttgtaaaagtgtgacagtggagaacttagttgtaagactgtgacagtggagatcacatttgta 10300

lcl|sc4wpfr_307 aaactgtgttatctggaccacaagctgtagaagggtttaggtgagaaataacagaagcaagagtggcgtagatatctagcagtgaattaaagtgaatggg 10400

lcl|sc4wpfr_307 ataataagaagagtcattagattcaagagattaagaggtttaaaataaaagttgtttgcaaatagttctgattgagagattaaatgcttgcttttattaa 10500

lcl|sc4wpfr_307 tgacattgtgtttttttattgctaaaaactgtctgataccaaaaaagtgtcagtttttagcattaataaaaagaaattctctagagagtttcatcttttc 10600

lcl|sc4wpfr_307 ttgtgaaaaagatgaaaaattgctagttagttagaaatagcagctatactagaatgtaaaaaccaagggtagattgagacttgacttgaaacaagaagga 10700

lcl|sc4wpfr_307 ataaaagtaattatgctttaatctagaagattataaaggaggaacaactttttagcacacaaagacattagcccaaatactatagcaatgaaaatcataa 10800

lcl|sc4wpfr_307 aatcctgtgagtcccagtcagctttatgatactaataaaaataggccacagcttctctaaaattttctaaagtcttctttttttaaaaatcttctcttaa 10900

lcl|sc4wpfr_307 ttcctctattttcctctaaaaagttagaaatgtgatcctaatttttctttaacttctcttccaacccccttcttgcatcatttcttgatggtagtaaatt 11000

lcl|sc4wpfr_307 gttaaaaatacccattcttggtcttcaaaactggttaacatcgaaaaggacattgggttgtaaaaaattgctttaacaaatccatgtcattaaacataaa 11100

lcl|sc4wpfr_307 aaatggcattattgacccaaactctacaagtgtggatacatggatgttaaacctatttttttgaaaaaaatcctctaaaatcctctatttttattcaaaa 11200

lcl|sc4wpfr_307 ttttctagttttgcaaaaaaattcttctaaattagatgcaaaatctcctttaaaatcctttaatgtactagtatgaagatttttgaatgcactcatgaat 11300

lcl|sc4wpfr_307 ttttgtgtactctttcgaatgtactcttacaaatttttaatgaaagtttttaatgcacgattataaaagttcttatgaaaattttatgtggccaccctga 11400

lcl|sc4wpfr_307 agaagtgtgacaccaaatgcactaacaggaacaccttccatgctcaacagcactgttatttgatattttatataacttactctgatattttatagctgtt 11500

lcl|sc4wpfr_307 gatagtatcagcagctaaaccatagttttcgagaaatctagctaccagcttcagaaccattaaatttagttttagaccttaccttcattaggagataata 11600

lcl|sc4wpfr_307 aaaagagtttctgctactaacacgcaacaaacagagtcaagaagatctagtgttcatcatcttttgctaccacaagcaacaaatagattcaacaagatct 11700

lcl|sc4wpfr_307 agtgttcatcatcctatacaggaaataaagaataaaaatgaattgtttttaatagaattttactttttaccaagtgcaccaaatcttttaataataattt 11800

lcl|sc4wpfr_307 aagttccatctgttaagtatctgttaagttcgatctttagattaataattattatttgcttattcacaatgattttttatatgcttgacctgtattaaat 11900

lcl|sc4wpfr_307 ttttcatatgcttgacctgtatcaaatctttaatatacttgacctgtatcaaatctttaatatacttgacagtgtatcaaatcttttatatgcttggcct 12000

lcl|sc4wpfr_307 gtatcaaatctttcatatgcttgacctgtattaaatttttcatatgcttgacctgtatcaaatcttctttcaatttattgtaaaatttggtttaaatttt 12100

lcl|sc4wpfr_307 ctgattatcttttatgttcttacacttttaagttactcgctttgttttctttctttttgttatccttgacattcttgtatgctccattttgatgttattt 12200

lcl|sc4wpfr_307 tatatataaatgcttaagttttctttaccatactgttgttggtaatgttaaattactaccataccttatttaatttgaagaaagtaaaatcaaaaatttt 12300

lcl|sc4wpfr_307 ttcttttatgttttattagttactgtctatttgttttttatgtttaattagttactttctatttgtttttttattttttattagttactgtcgatttgtt 12400

lcl|sc4wpfr_307 agcttaaagttgacaatcaatacttaacaataacgtaatgttcacagcacttattaaaaatgcaatttttaacaacttaatctttagctatttactataa 12500

lcl|sc4wpfr_307 atatctcttaaaatagttttatatcctaaaaatagttttaggacaattgtatactgcaaggtttgtgcttaaattaagtaatatgttcccatattttgat 12600

lcl|sc4wpfr_307 gtaatatatttttcatcaaatgatatgttaaagtagatctattcccaaaaatgtcttttccaattttttatacatttcagactaaaatatcccctcctcc 12700

lcl|sc4wpfr_307 taactttaataaactctgggattgtcgtagatcacttttaatattattttaataaacattttttagataattgtaagcatttattttagattattatttt 12800

12810 12820 12830 12840 12850 12860 12870 12880 12890 12900

....|....|....|....|....|....|....|....|....|....|....|....|....|....|....|....|....|....|....|....|

**DMTF1_AEP L N F L N W S E T G G K K W N K F C D L E L I**

c21737_g1_i04 ----------------------------------------------------------------------------------------------------

HAEP_T-CDS_v02_6413 ------------------------------GTTAAATTTTTTGAATTGGTCTGAAACTGGTGGTAAAAAATGGAATAAATTTTGTGATTTAGAGCTTATC

lcl|Sc4wPfr_307 tcgtggcatatttttaaacttgtttcatagGTTAAACTTTTTGAATTGGTCTGAAACTGGTGGTAAAAAATGGAATAAATTTTGTGATTTAGAGCTTATC

12910 12920 12930 12940 12950 12960 12970 12980 12990 13000

....|....|....|....|....|....|....|....|....|....|....|....|....|....|....|....|....|....|....|....|

**DMTF1_AEP N K**

c21737_g1_i04 ----------------------------------------------------------------------------------------------------

HAEP_T-CDS_v02_6413 AATAA----------------------------------------------------------------------------------------------

lcl|Sc4wPfr_307 AGTAAgtaagtggattatgtttgctctttaactttttaataatctgactgtgagtgaggtaaatatttttatgacctgactgtgaggtacacttaaaata

lcl|sc4wpfr_307 aagaatttaaagaaatatctaaaaaaacatttttttgaacattagtaatatttaaagtcaatttatttacagagttatagtacccacatcagctgtcagt 13100

lcl|sc4wpfr_307 cctgcttagccaccatattagtggagccatgtttatttttacccctgatttaaagtatcatgttgacagcaaatagtttctactctatgatatcaaaaca 13200

lcl|sc4wpfr_307 gagaagtgtgacaagtattttatcaggttgagcaggtttttactttaatcaaaagaaatatccacttctgttactttttgttttttttaattcactatca 13300

lcl|sc4wpfr_307 atgactataccaatattggaataacaaatcatttttcttgcagcgtttgcagcataaatcaaactatatacagaaactaatactatatatactatataaa 13400

lcl|sc4wpfr_307 ctatatactatataaactatatactatataaactatatactatataaactatatactatataaactatatactatatatctaagcataaacatacatatg 13500

lcl|sc4wpfr_307 tttggttcaaaaaatatgtttttttaaacatcttttaaatgttaaaaaatttttttttgcagaccaaatgcaggtgggcatgttaaagagatgtgaagtt 13600

lcl|sc4wpfr_307 ttttacacctaaagtagttttgcatgttttacttcagaatatgatttaaaaactgctgctgttatatgtagaaaactatctattatatgtagaaactgct 13700

lcl|sc4wpfr_307 attatcatactatatctgttatatgtagaaactgctattatcatacgtagtaaactgctgctgttatatgtagaaaactgctgttatcatacgtagtaaa 13800

lcl|sc4wpfr_307 ctgctgctgttatatgtagaaaactagaaattagaaatagtttaggaaattgaaagtcaatttataaaagaatgttttctcaattttttgctctctatga 13900

lcl|sc4wpfr_307 aagagattttcataaaaacattgtatattaacatgaaaagttgtatattttttatacagtttataaatgtttaaattaattgcaacattttgtaaaaaat 14000

lcl|sc4wpfr_307 cttcgaaagttaaaaaaaggtaaatataaaaaaattaacttgatgcaaactgttttgacaactatttgagtatgcgttggatgtaagaatgtaaggatgt 14100

lcl|sc4wpfr_307 tcggatgtttgtaagaggtttgacagatgtctctatgaaaatgcatgccaactgtttaaaagctaaagtttctatgatggctaccagcgctcaaaataag 14200

lcl|sc4wpfr_307 ggtcaatcaacaatcaatgatcggctatttttagggtaatttcgaaaaattcaaggaaatataacctgtttttttgaaaaaattacatttgatcggcaag 14300

lcl|sc4wpfr_307 ctttgaaaaactatttcgagcgctgggctacgattattagagtaataattgtgtaattttaatactttgaaaaattttaaattaatgttaggaagttaat 14400

lcl|sc4wpfr_307 aatgaaatcaccttcttttaagtaatttttttttaaactaataataatagaaatatatttcttatttaaagaaataaatttaccaccccacaatttgttt 14500

lcl|sc4wpfr_307 cttgttattttaagaaataaattttttaaacttttaaacaatggtcttacgattttgctctatattcaagattacaacttttcaattgttcttaattacg 14600

lcl|sc4wpfr_307 tcagtgttaattaaaagagtgggtttgctcaaaacgttttaaacaaatcctcttttttaaaaacattgatgtcagttttttagtatttgttttctattac 14700

14710 14720 14730 14740 14750 14760 14770 14780 14790 14800

....|....|....|....|....|....|....|....|....|....|....|....|....|....|....|....|....|....|....|....|

**DMTF1_AEP I G N L N V I N E S K I D W I S L A K D W C**

c21737_g1_i04 ---------------------------------------------------AGTCTGGCTAAAGATTGGTGCAGGTCTAACTAATTTTAGTTTAGTTTAA

HAEP_T-CDS_v02_6413 -----AATTGGAAATTTAAATGTTATCAATGAGTCAAAAATTGATTGGATCAGTCTGGCTAAAGATTGGTGC----------------------------

lcl|Sc4wPfr_307 attagAATTGGAAATTTAAATGTTATCAATGAGTCAAAAATTGATTGGAGCAATCTGGCTAAAGATTGGTGCAGgtctaactaattttagtttaaagcta

14810 14820 14830 14840 14850 14860 14870 14880 14890 14900

....|....|....|....|....|....|....|....|....|....|....|....|....|....|....|....|....|....|....|....|

**DMTF1_AEP S V R S P Q W L K H R W**

c21737_g1_i04 AGCTATTTAATTGTTTTTTTTATTTAAGTCTTTTTTCTCTAAGTTTGCATTAAGGTATTTTTCAGTGTTCGTTCACCACAGTGGTTAAAACACAGATGGC

HAEP_T-CDS_v02_6413 ---------------------------------------------------------------AGTGTTCGTTCACCACAGTGGTTAAAACACAGATGGC

lcl|Sc4wPfr_307 tttaattttttttta-----------tgtcttttttctctaagtttacattgaggtatttttcaGTGTTCGTTCACCACAGTGGTTAAAACACAGATGGC

#### Hydra ms-DMTF1

14910 14920 14930 14940 14950 14960 14970 14980 14990 15000

....|....|....|....|....|....|....|....|....|....|....|....|....|....|....|....|....|....|....|....|

**DMTF1_AEP H G I K K H V P N Y Q H L T**

c21737_g1_i04 ATGGAATTAAAAAACATGTGCCAAATTATCAACATTTAAC------------------------------------------------------------

HAEP_T-CDS_v02_6413 ATGGAATTAAAAAACATGTGCCAAATTATCAACATTTAAC------------------------------------------------------------

lcl|Sc4wPfr_307 ACGGAATCAAAAAACATGTACCAAATTATCAACATTTAACattaaatggtaagcaatacttatttataaataatataaataatataaataataaataaaa

lcl|sc4wpfr_307 aatataaataataaagttagatatttttaactttattatttataaaataatttttataaacaaaatagcaagcacagtttgtttataaaaattttgtaaa 15100

lcl|sc4wpfr_307 taatgtttttttttttaaaaagattaaaattgataaaatattaattataaaaataaataaaatgctatttatctaattaattgataaaaaatataaaact 15200

lcl|sc4wpfr_307 tttaaagttttttgaaacctaaatttataatacataatagttgtacatatttgatttattaaatgtttactataaattaaatattgtgtgattcattttt 15300

15310 15320 15330 15340 15350 15360 15370 15380 15390 15400

....|....|....|....|....|....|....|....|....|....|....|....|....|....|....|....|....|....|....|....|

**DMTF1_AEP L N N I I G F L Q T N Y V K E V I H K L N E H V S V R N N Q**

c21737_g1_i04 ----------GTTAAATAACATAATAGGTTTTCTGCAAACCAATTATGTTAAAGAAGTTATTCATAAATTAAATGAACATGTGTCAGTCAGAAATAATCA

HAEP_T-CDS_v02_6413 ----------GTTAAATAACATAATAGGTTTTCTGCAAACCAATTATGTTAAAGAAGTTATTCATAAATTAAATGAACATGTGTCAGTCAGAAATAATCA

lcl|Sc4wPfr_307 attattttttGTTGGTAGACATAATAGGTTTTTTGCAAACCAATTATGTTAAAGAAGTTATTCATAAATTAAATGAACATGTGTCAGTCAGAAATAATCG

15410 15420 15430 15440 15450 15460 15470 15480 15490 15500

....|....|....|....|....|....|....|....|....|....|....|....|....|....|....|....|....|....|....|....|

**DMTF1_AEP D E L V L T L N A Q S L V Q E F Q I E A N D S Q S K S F I N**

c21737_g1_i04 AGATGAACTCGTGCTTACATTGAATGCTCAATCATTGGTTCAAGAGTTTCAAATAGAAGCT---------AATGATTCACAGTCAAAATCTTTTATTAAC

HAEP_T-CDS_v02_6413 AGATGAACTCGTGCTTACATTGAATGCTCAATCATTGGTTCAAGAGTTTCAAATAGAAGCT---------AATGATTCACAGTCAAAATCTTTTATTAAC

lcl|Sc4wPfr_307 AGATGAACTCGTGCTTACGTCGAATGATCAATCATTGGTTCAAGAGTTTCAAAAAGAAGCTtcacactcaAAATCTTCACAGTCAAAATCTTTTATTAAC

15510 15520 15530 15540 15550 15560 15570 15580 15590 15600

....|....|....|....|....|....|....|....|....|....|....|....|....|....|....|....|....|....|....|....|

**DMTF1_AEP K N Q L E I L**

c21737_g1_i04 AAAAATCAACTTGAAATATTAG------------------------------------------------------------------------------

HAEP_T-CDS_v02_6413 AAAAATCAACTTGAAATATTAG------------------------------------------------------------------------------

lcl|Sc4wPfr_307 AAAAATCAACTTGAAATATTAGgtacagtgtttaacatttaaaaataatttttatatatgttaaatatttgacaaactttttaaaattctgaaaaatatc

lcl|sc4wpfr_307 tgtaaaatattaatatagtattaatttaatctaaacatttttttttcttttatttaaccatattaataaaattaacaacagttcataataaatttacaga 15700

lcl|sc4wpfr_307 aatttattcggcgtcatttatattgattacacactagtcaatggagtgacacctataaacataaaaagcacaaacgaacaaaaaaataaaaaagacagaa 15800

lcl|sc4wpfr_307 caagaaaaataaaataaaaaggagacagaaaaaaaatatttagataaataaaataaaaaatcaaatataaaaagaaatacattaataataatattaataa 15900

lcl|sc4wpfr_307 taacattatatattaataataataacaacaaacttattattaaaaaaaatcaataataaattattgatttttttttttatttgtataaaagtgtttgttg 16000

lcl|sc4wpfr_307 aaatcggttttgacaaaaaaactatccaaaagatagctattgtaataaaatgtttttctctgcggtatctatagtaacattatcagatacattatcataa 16100

lcl|sc4wpfr_307 gcactttagtttatgtttttgctatggttaatgaatataattaatgtaatacttaagtgatttattcagtgttatttaatttaactaccacatgttttta 16200

lcl|sc4wpfr_307 aagaagagtttatgttatgataaataaaatcaaagataataaagaagtacaatgaacacttaaaaaaaaagaaaatctacactgaaatcttaaaaaaaaa 16300

lcl|sc4wpfr_307 taagtacactgaatgcttgaattgcaattctaatgtcaagacagtgacaagtatttatacagcaactttttttgtctttttaaataaaagtaacttcaaa 16400

lcl|sc4wpfr_307 tgaaaattttatatggaaaccgttttgaaaatataataaaagtgttttaaataaaaattcaacaggagaccctaaggtaggtttctagcaaccaaacttc 16500

lcl|sc4wpfr_307 tgattcttcagaagtttagatgctaagaagttatttaaaaaaaagatttaggtgtcaaaaagaaagaagcaagaaacacaaagaaaaagtaatcttcaaa 16600

lcl|sc4wpfr_307 gaaaaatttatttttctcttcgactttttccttaagaaacactctaccttaaaaacactctaccttaagaaacactctacctgaagaagcactctacctt 16700

lcl|sc4wpfr_307 aagaaacactctacctcaagaagcactccaccttaagaaacactctaccttaagaaatactctaccttaaaaatactctacctttaaaacactctacctt 16800

lcl|sc4wpfr_307 aagaaacactctaccttagtgtgaaaaaaagatgaatagagtgacaacttgttagcaattcaatgagaaatatgcttttcttcttcaaccaatgaaaagg 16900

lcl|sc4wpfr_307 aacaaaaagctaattaaataatgattgtaaatatttgcaagctcaaaactaaaattattatcatgtatttaccggtgtagttaaaggtattccttgtagg 17000

lcl|sc4wpfr_307 atgtggtccaataatagaaccaatagtatacaatagtgggtgtctaagtgtgggtccaataatagaactaatatatactttatataatatataaagtata 17100

lcl|sc4wpfr_307 ctactgtatatatattatataatagtatattttatattttatcaaatagtaacaacggtatacaaatataacttgtaatgtacaactatcacttgtaaag 17200

lcl|sc4wpfr_307 tacgtccaattaaaaaaagtgacaaaatcaatatgcttttgataattaatgtaatttttgataatttgttataaaaaacatttaagtaaagtaaaatcat 17300

lcl|sc4wpfr_307 tatgatcatgagaatagtcaccttagttactgtatttccatgagacatgtccagatgtacaaaggtactaaatgacccttggtacaagattaaatagcat 17400

lcl|sc4wpfr_307 agcctcataaaaaataacatttactaaaaaaggccacattaggaaattcttttttttttaattgcatctagtaaccagtggtgcaaacaagttacttgat 17500

lcl|sc4wpfr_307 gaactcaaagaagggtgttttctttgaattttctaaatttaaatgaaggatttaaaacaaaagtaattccaacattttactattataaaaaattatactc 17600

lcl|sc4wpfr_307 aatttataacttgattataactacaggttataacttgctacctcctttaacatattaaattagataaaaaaatttatcaactacttgattaagcaacata 17700

lcl|sc4wpfr_307 gtaatgaacttaatgttgtagggcagaacttacacccttaattcgtaagcacctttgatcctttaactccatacttacataacttattaaagaggactgt 17800

lcl|sc4wpfr_307 caaccagcatgttataaatattgtatcttcaaaattaattgcgtttttgatagagaagagaccactattaaatttgtttttaaaaagtttgattttgaca 17900

lcl|sc4wpfr_307 acaaaaagaaatttttttacttttacaaggaacctcacatctttaaaatttttttcgcttatgtcattttgtaactaaactatttccctcttgtatgctt 18000

lcl|sc4wpfr_307 ttggtaagctatagatgcatctaatatgacaaaaaaagaatttttgtaaaaatgaagtgggttagtacccttgatcctaatgcatttacgtccctggaag 18100

lcl|sc4wpfr_307 taccttgctcaatttgtttctccgagcaacggccttattcgtcaaggttcgtgttttggaggtaaagagtttaaagaaggttgtaaccaaaatgaagtag 18200

18210 18220 18230 18240 18250 18260 18270 18280 18290 18300

....|....|....|....|....|....|....|....|....|....|....|....|....|....|....|....|....|....|....|....|

**DMTF1_AEP G Q V N**

c21737_g1_i04 ------------------------------------------------------------------------------------------GTCAAGTAAA

HAEP_T-CDS_v02_6413 ------------------------------------------------------------------------------------------GTCAAGTTAA

lcl|Sc4wPfr_307 cctcctcgactgtagtggccttcttggccttgtggaggtgaataacattaataaaaaataatctgtaaatctttatatttgattttttagAACAAGTAAA

18310 18320 18330 18340 18350 18360 18370 18380 18390 18400

....|....|....|....|....|....|....|....|....|....|....|....|....|....|....|....|....|....|....|....|

**DMTF1_AEP D P S A L I P L S S Q E D M T H G G F T I L F ***

c21737_g1_i04 CGACCCATCTGCTTTAATACCCCTTTCATCTCAAGAAGATATGACGCATGGGGGTTTCACGATTTTATTTTAGAAAAAAGTATTGATCGATTTAACTGCT

HAEP_T-CDS_v02_6413 CGACCCATCTGCTTTAATACCCCTTTCATCTCAAGAAGATATGACGCATGGGGGTTTCACGATTTTATTTTAGAAAAAAGTATTGATCGATTTAACTGCT

lcl|Sc4wPfr_307 CGATCCATCTACTTTAATACCCCTTTCATCTCAAGAAGATACGACGCATGGGAGTTTCACAATTTTATTTTAGAAAAAAGTATTTATCGATTTAACTGCT

18410 18420 18430 18440 18450 18460 18470 18480 18490 18500

....|....|....|....|....|....|....|....|....|....|....|....|....|....|....|....|....|....|....|....|

c21737_g1_i04 GAAGGATATATTTTTTCATATATATATATATACATACAT-------------------------------------------------------------

HAEP_T-CDS_v02_6413 GAAGGATATATTTTTTCATATATATATATATACATACATATATATA------------------------------------------------------

lcl|Sc4wPfr_307 GAAGGATATATTTTTTCATATATATATATATATATATATATATATATATATATATTTTCATATGTATATATATATATATATATATATATATATATATATA

18510 18520 18530 18540 18550 18560 18570 18580 18590 18600

....|....|....|....|....|....|....|....|....|....|....|....|....|....|....|....|....|....|....|....|

c21737_g1_i04 -----------------------------------------------------AGAAACTATTTATTAAATCTATTGCATTATTTTAAATCTGTGATTTG

HAEP_T-CDS_v02_6413 ----------------------------------------------CATACATAGAAACTATTTATTAAATCTATTGCATTATTTTAAATCTGTGATTTG

lcl|Sc4wPfr_307 TATATATATTTTTTTTTTCATATGTATATATATACATACATACATACATACATAGAAACAATTTATTAAATCTATTGCATTATTTTAAATCTGTGGTTTT

18610 18620 18630 18640 18650 18660 18670

....|....|....|....|....|....|....|....|....|....|....|....|....|....|....|..

c21737_g1_i04 GTTGTAATATATATACATAAACACTCA--------------------------------------------------

HAEP_T-CDS_v02_6413 GTTGTAATATATATACATAAACACTCATTTGTAAGTTTATTTTTGTAAATAAGTTATTTTATTTGATTAGTAA----

lcl|Sc4wPfr_307 GTTGTAATAT----ACATGAACACTCATTTCTAAGTTTATTTTTGTAAATAAGTTATTTTATTTAGTTAGTAAAATT

### S6 Fig. Alignment of the *Hydra* transcriptomic and genomic sequences corresponding to *ms-DMTF1.*

Alignment of the *ms-DMTF1* sequences identified in *AEP* transcriptomes (*c21737_g1_i4, HAEP_T-CDS_v02_6413*) and *Hm-105* genome (*lcl|Sc4wPfr_307)* see **S2 Table**. The putative *DMTF1* coding sequences are boxed. The microsatellite region (*ms-DMTF1*) is located in the 3’ untranslated region (3’UTR), highlighted in yellow. The primer sequences are underlined and highlighted in grey.
