# Supplementary material for: The polymorphism of Hydra microsatellite sequences provides strain-specific signatures
Source: PLoS One. 2020 Sep 28;15(9):e0230547. doi: 10.1371/journal.pone.0230547 (PMC7521734; doi:10.1371/journal.pone.0230547)
Supplement: S7 Fig — (DOCX) [file pone.0230547.s009.docx]

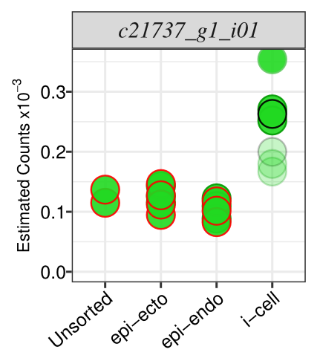

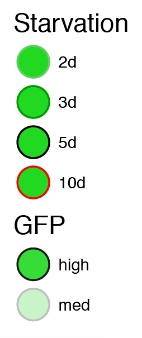

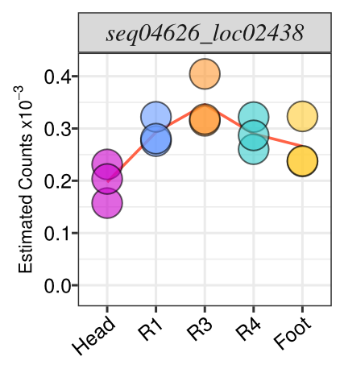

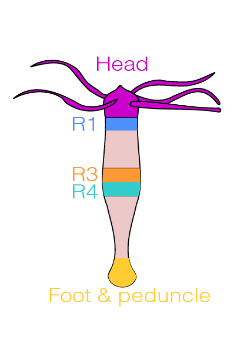

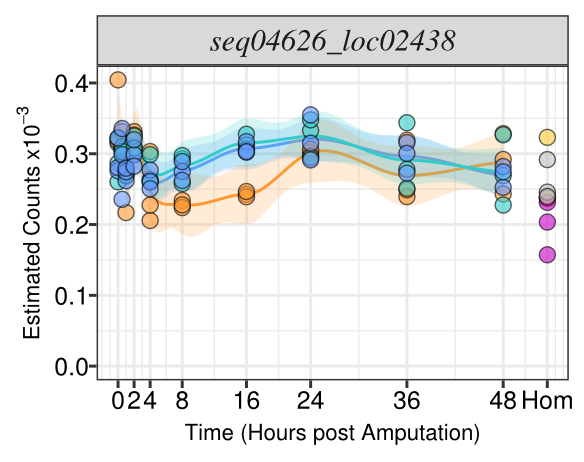

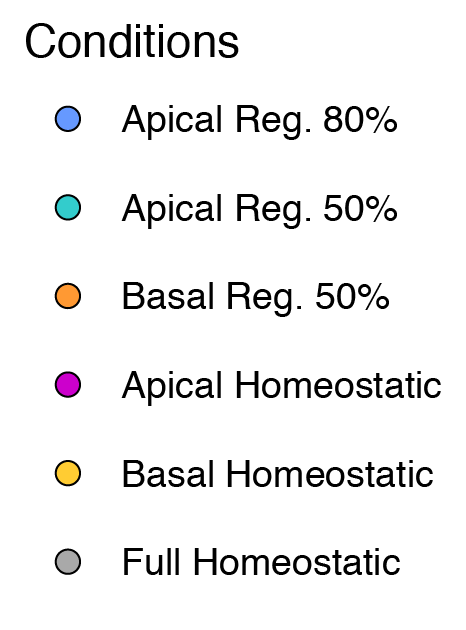


### S7 Fig. Expression profile of the *Hydra* *DMTF1* gene.

(Upper left) Expression levels of *DMTF1* in the three stem cell populations, either epithelial from the epidermis (epi-ecto) or epithelial from the gastrodermis (epi-endo), or interstitial (i-cell, left panel) ([HydrATLAS c21737_g1_i01](https://hydratlas.unige.ch/blast/get_sequences.cgi?alignment_seq_1=c21737_g1_i04%7CHydra+vulgaris+AEP&alignment_seq_2=c21737_g1_i01%7CHydra+vulgaris+AEP&alignment_seq_3=c21737_g1_i02%7CHydra+vulgaris+AEP&alignment_seq_4=c21737_g1_i03%7CHydra+vulgaris+AEP&alignment_seq_5=seq04629_loc02438%7CHydra+vulgaris+Jussy&alignment_seq_6=seq04627_loc02438%7CHydra+vulgaris+Jussy&alignment_seq_7=seq04626_loc02438%7CHydra+vulgaris+Jussy&alignment_seq_8=seq04625_loc02438%7CHydra+vulgaris+Jussy&hit_count=8&database=db%2FHv_AEP_transcriptome.fasta)). Note the enrichment in the i-cell fraction. (Upper right): Expression levels of *DMTF1* at five positions along the body axis in intact animals. (Lower panel) Expression levels of *DMTF1* at 9 distinct time-points after mid-gastric bisection or after decapitation (blue dots) as depicted on the schemes (see [HydrATLAS seq04626_loc02438](https://hydratlas.unige.ch/blast/get_sequences.cgi?hit_count=1&alignment_seq_1=seq04626_loc02438&database=full_database)).
