# Supplementary figures and images for: The polymorphism of Hydra microsatellite sequences provides strain-specific signatures
Source: PLoS One. 2020 Sep 28;15(9):e0230547. doi: 10.1371/journal.pone.0230547 (PMC7521734; doi:10.1371/journal.pone.0230547)

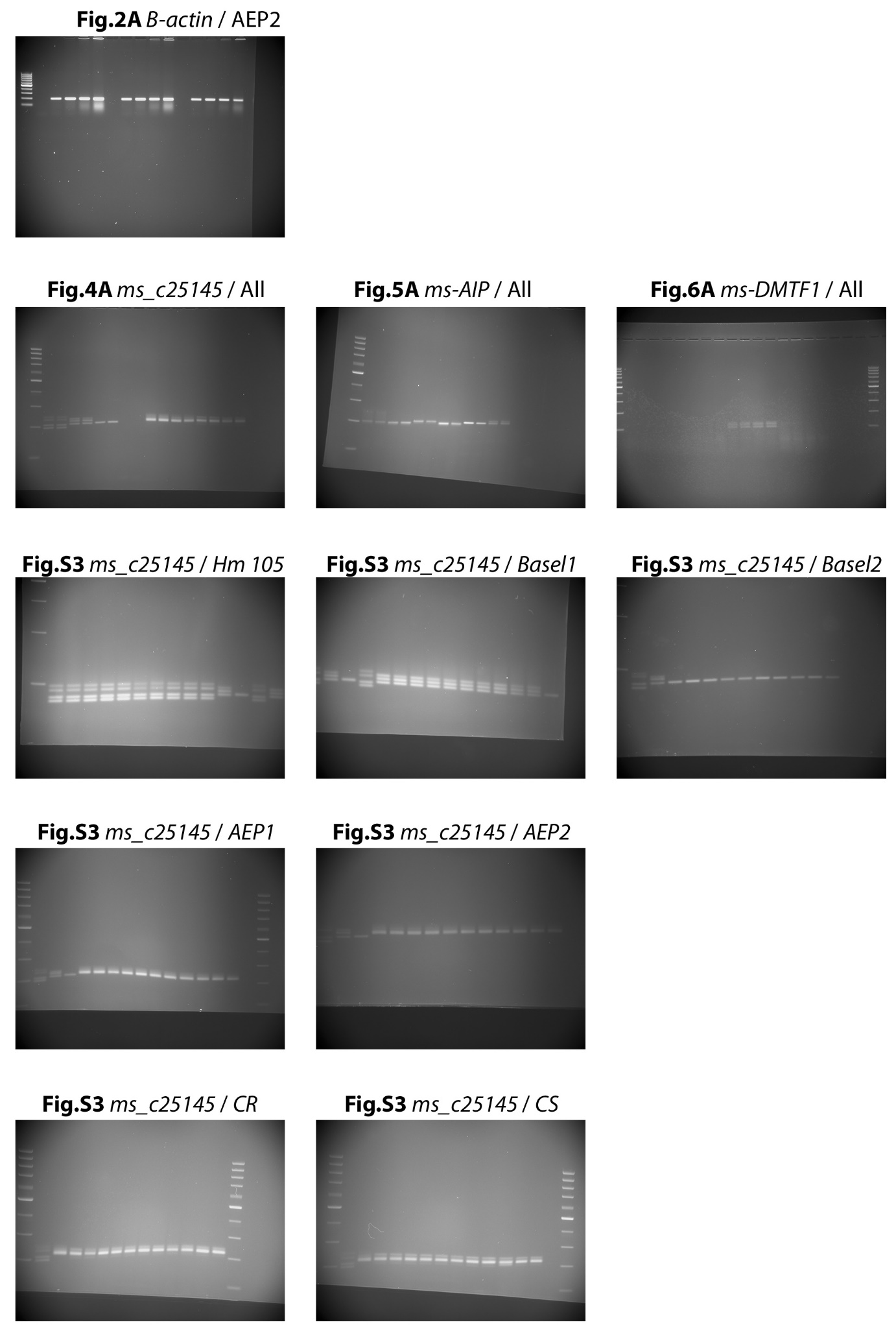


### S8 Fig. Intact gels corresponding to the data shown in the panels from Figs 2A, 4A, 5A, 6A and S3 Fig.

Supplement: S8 Fig — (DOCX) [file pone.0230547.s010.docx]
